# Assisted Reproductive Technology Surveillance — United States, 2016

**DOI:** 10.15585/mmwr.ss6804a1

**Published:** 2019-04-26

**Authors:** Saswati Sunderam, Dmitry M. Kissin, Yujia Zhang, Suzanne G. Folger, Sheree L. Boulet, Lee Warner, William M. Callaghan, Wanda D. Barfield

**Affiliations:** 1 Division of Reproductive Health, National Center for Chronic Disease Prevention and Health Promotion, CDC; 2 Emory University School of Medicine, Atlanta, Georgia

## Abstract

**Problem/Condition:**

Since the first U.S. infant conceived with assisted reproductive technology (ART) was born in 1981, both the use of ART and the number of fertility clinics providing ART services have increased steadily in the United States. ART includes fertility treatments in which eggs or embryos are handled in the laboratory (i.e., in vitro fertilization [IVF] and related procedures). Although the majority of infants conceived through ART are singletons, women who undergo ART procedures are more likely than women who conceive naturally to deliver multiple-birth infants. Multiple births pose substantial risks for both mothers and infants, including obstetric complications, preterm delivery (<37 weeks), and low birthweight (<2,500 g). This report provides state-specific information for the United States (including the District of Columbia and Puerto Rico) on ART procedures performed in 2016 and compares birth outcomes that occurred in 2016 (resulting from ART procedures performed in 2015 and 2016) with outcomes for all infants born in the United States in 2016.

**Period Covered:**

2016.

**Description of System:**

In 1995, CDC began collecting data on ART procedures performed in fertility clinics in the United States as mandated by the Fertility Clinic Success Rate and Certification Act of 1992 (FCSRCA) (Public Law 102–493 [October 24, 1992]). Data are collected through the National ART Surveillance System (NASS), a web-based data collection system developed by CDC. This report includes data from 52 reporting areas (the 50 states, the District of Columbia, and Puerto Rico).

**Results:**

In 2016, a total of 197,706 ART procedures (range: 162 in Wyoming to 24,030 in California) with the intent to transfer at least one embryo were performed in 463 U.S. fertility clinics and reported to CDC. These procedures resulted in 65,964 live-birth deliveries (range: 57 in Puerto Rico to 8,638 in California) and 76,892 infants born (range: 74 in Alaska to 9,885 in California). Nationally, the number of ART procedures performed per 1 million women of reproductive age (15–44 years), a proxy measure of the ART use rate, was 3,075. ART use rates exceeded the national rate in 14 reporting areas (Connecticut, Delaware, the District of Columbia, Hawaii, Illinois, Maryland, Massachusetts, New Hampshire, New Jersey, New York, Pennsylvania, Rhode Island, Utah, and Virginia). ART use exceeded 1.5 times the national rate in nine states, including three (Illinois, Massachusetts, and New Jersey) that also had comprehensive mandated health insurance coverage for ART procedures (i.e., coverage for at least four oocyte retrievals).

Nationally, among ART transfer procedures for patients using fresh embryos from their own eggs, the average number of embryos transferred increased with increasing age (1.5 among women aged <35 years, 1.7 among women aged 35–37 years, and 2.2 among women aged >37 years). Among women aged <35 years, the national elective single-embryo transfer (eSET) rate was 42.7% (range: 8.3% in North Dakota to 83.9% in Delaware).

In 2016, ART contributed to 1.8% of all infants born in the United States (range: 0.3% in Puerto Rico to 4.7% in Massachusetts). ART also contributed to 16.4% of all multiple-birth infants, including 16.2% of all twin infants and 19.4% of all triplets and higher-order infants. ART-conceived twins accounted for approximately 96.5% (21,455 of 22,233) of all ART-conceived infants born in multiple deliveries. The percentage of multiple-birth infants was higher among infants conceived with ART (31.5%) than among all infants born in the total birth population (3.4%). Approximately 30.4% of ART-conceived infants were twins and 1.1% were triplets and higher-order infants.

Nationally, infants conceived with ART contributed to 5.0% of all low birthweight (<2,500 g) infants. Among ART-conceived infants, 23.6% had low birthweight compared with 8.2% among all infants. ART-conceived infants contributed to 5.3% of all preterm (gestational age <37 weeks) infants. The percentage of preterm births was higher among infants conceived with ART (29.9%) than among all infants born in the total birth population (9.9%).

The percentage of ART-conceived infants who had low birthweight was 8.7% among singletons, 54.9% among twins, and 94.9% among triplets and higher-order multiples; the corresponding percentages among all infants born were 6.2% among singletons, 55.4% among twins, and 94.6% among triplets and higher-order multiples. The percentage of ART-conceived infants who were born preterm was 13.7% among singletons, 64.2% among twins, and 97.0% among triplets and higher-order infants; the corresponding percentages among all infants were 7.8% for singletons, 59.9% for twins, and 97.7% for triplets and higher-order infants.

**Interpretation:**

Multiple births from ART contributed to a substantial proportion of all twins, triplets, and higher-order infants born in the United States. For women aged <35 years, who typically are considered good candidates for eSET, on average, 1.5 embryos were transferred per ART procedure, resulting in higher multiple birth rates than could be achieved with single-embryo transfers. Of the four states (Illinois, Massachusetts, New Jersey, and Rhode Island) with comprehensive mandated health insurance coverage, three (Illinois, Massachusetts, and New Jersey) had rates of ART use >1.5 times the national average. Although other factors might influence ART use, insurance coverage for infertility treatments accounts for some of the difference in per capita ART use observed among states because most states do not mandate any coverage for ART treatment.

**Public Health Action:**

Twins account for almost all of ART-conceived multiple births born in multiple deliveries. Reducing the number of embryos transferred and increasing use of eSET, when clinically appropriate, could help reduce multiple births and related adverse health consequences for both mothers and infants. Because multiple-birth infants are at increased risk for numerous adverse sequelae that cannot be ascertained from the data collected through NASS alone, long-term follow-up of ART infants through integration of existing maternal and infant health surveillance systems and registries with data available from NASS might be useful for monitoring adverse outcomes.

## Introduction

Since the birth of the first U.S. infant conceived with assisted reproductive technology (ART) in 1981, use of advanced technologies to overcome infertility has increased, as has the number of fertility clinics providing ART services and procedures in the United States (*1*). In 1992, Congress passed the Fertility Clinic Success Rate and Certification Act (FCSRCA) (Public Law 102–493 [October 24, 1992]), which requires that all U.S. fertility clinics performing ART procedures report data to CDC annually on every ART procedure performed. CDC initiated data collection in 1995 and in 1997 published the first annual ART Fertility Clinic Success Rates Report (*2*). Two reports are produced annually (ART Fertility Clinic Success Rates Report and ART National Summary Report) (*1*,*3*). These reports present multiple measures of success for ART, including the percentage of ART procedures and transfers that result in pregnancies, live-birth deliveries, singleton live-birth deliveries, and multiple live-birth deliveries.

Although ART helps millions of persons achieve pregnancy, ART is associated with potential health risks for both mothers and infants. Because multiple embryos are transferred in most ART procedures, ART often results in multiple-gestation pregnancies and multiple births (*4*–*11*). Risks to the mother from a multiple-birth pregnancy include higher rates of caesarean delivery, maternal hemorrhage, pregnancy-related hypertension, and gestational diabetes (*12*,*13*). Risks to the infant include preterm birth, low birthweight, death, and greater risk for birth defects and developmental disability (*4*–*16*). Further, singleton infants conceived with ART might have higher risk for low birthweight and prematurity than singletons not conceived with ART (*17*). However, recent research suggests that this higher risk might be associated with multiple embryo transfers resulting in singleton births because the higher risk was observed among patients administered ART who were not good candidates for elective single-embryo transfer (eSET) and had multiple embryos transferred (*18*).

This report was compiled from data about ART procedures performed in 2016 and reported to CDC’s Division of Reproductive Health. Data on the use of ART are presented for residents of each U.S. state, the District of Columbia, and Puerto Rico. Data also are reported on outcomes for infants born in 2016 resulting from ART procedures performed in 2015 and 2016. The report also examines the contribution of ART to selected outcomes (e.g., multiple-birth infants, low birthweight infants, preterm infants, and small for gestational age infants) and compares outcomes among ART-conceived infants with outcomes among all infants born in the United States in 2016.

## Methods

### National ART Surveillance System

In 1995, CDC initiated data collection of ART procedures performed in the United States. ART data are obtained from all fertility clinics in the United States through the National ART Surveillance System (NASS), a web-based data collection system developed by CDC (https://www.cdc.gov/art/nass/index.html). Clinics that are members of the Society for Assisted Reproductive Technology (SART) can report their data to NASS through SART and enter their data directly into NASS. Clinics that are not members of SART can enter their data directly into NASS. All clinics must verify the accuracy of the data they reported in the clinic table in the annual ART Fertility Clinic Success Rates Report before finalizing submission to NASS. The data then are compiled by CDC contractor Westat Inc., a statistical survey research organization, and reviewed for accuracy by both CDC and Westat. In 2016, a small proportion of clinics (8%) did not report their data to CDC and are listed as nonreporting clinics in the 2016 ART Fertility Clinic Success Rates Report, as required by FCSRCA. Because nonreporting clinics tend to be smaller on average than reporting clinics, NASS is estimated to contain information on 97% of all ART procedures in the United States (*1*).

Data collected include patient demographics, medical history, and infertility diagnoses; clinical information pertaining to the ART procedure type; and information regarding resultant pregnancies and births. The data file contains one record per ART procedure (or cycle of treatment) performed. Because ART providers typically do not provide continued prenatal care after a pregnancy is established, ART clinics collect information on live births for all procedures from patients and physicians.

### ART Procedures

ART includes fertility treatments in which eggs or embryos are handled in a laboratory (i.e., in vitro fertilization [IVF], gamete intrafallopian transfer, and zygote intrafallopian transfer). More than 99% of ART procedures performed are IVF. Because an ART procedure consists of multiple steps over an interval of approximately 2 weeks, a procedure often is referred to as a cycle of treatment. An ART cycle usually begins with drug-induced ovarian stimulation. If eggs are produced, the cycle progresses to the egg-retrieval stage, which involves surgical removal of the eggs from the ovaries. After the eggs are retrieved, they are combined with sperm in a laboratory during the IVF procedure. For certain IVF procedures (66.0% in 2016) (*1*), a specialized technique (intracytoplasmic sperm injection) is used where a single sperm is injected directly into the egg. If successful fertilization occurs, the most viable embryos (i.e., those that appear morphologically most likely to develop and implant) are selected for transfer back into the uterus. If an embryo implants in the uterus, a clinical pregnancy is diagnosed by the presence of a gestational sac detectable by ultrasound. Most pregnancies will progress to a live-birth delivery, defined as the delivery of one or more live-born infants; however, some result in pregnancy loss (*19**,**20*). ART does not include treatments in which only sperm are handled (i.e., intrauterine insemination) or procedures in which a woman is administered drugs to stimulate egg production without the intention of having eggs retrieved.

ART procedures are classified on the basis of the source of the egg (patient or donor) and the status of the eggs and embryos. Both fresh and thawed embryos can be derived from fresh or frozen eggs of the patient or donor. Patient and donor embryos can be created using sperm from a partner or donor. ART procedures involving fresh eggs and embryos include an egg-retrieval stage. ART procedures that use thawed eggs or embryos do not include egg retrieval because the eggs were retrieved during a previous ART procedure and either the eggs were frozen or fertilized and the resultant embryos were frozen until the current ART procedure. An ART cycle can be discontinued at any step for medical reasons or by patient choice.

### Birth Data for United States

Data on the total number of live-birth and multiple-birth infants in each reporting area in 2016 were obtained from U.S. natality files (*21**–**23*). The natality online databases report counts of live births and multiple births occurring within the United States to residents and nonresidents. The data are derived from birth certificates.

### Variables and Definitions

Data on ART outcomes and ART procedures are presented by patient’s residence (i.e., reporting area) at the time of treatment, which might not be the same as the location where the procedure was performed. If information on patient’s residence was missing, residence was assigned as the location where the procedure was performed (0.4% of procedures performed in 2016 and 0.2% of live-birth deliveries occurring in 2016). ART procedures performed in the United States among nonresidents who are non-U.S. citizens are included in NASS data (*24*); however, they are excluded from certain calculations because residency status is unknown. To protect confidentiality, table cells with values of 1–4 for ART-conceived infants and 0–9 for all infants are suppressed. Because of limited numbers, ART data from U.S. territories (with the exception of Puerto Rico) are not included in this report. In addition, estimates derived from cell values <20 in the denominator have been suppressed because they are unstable and estimates could not be calculated when the denominator was zero (e.g., preterm birth among triplets in reporting areas with no triplet births).

This report presents data on all procedures initiated with the intent to transfer at least one embryo, including procedures that used thawed frozen eggs for transfer. Cycles with the intent to freeze all eggs or embryos for future ART cycles were excluded. The number of ART procedures performed per 1 million women of reproductive age (15–44 years) was calculated (*25*). Data regarding population size were compiled on the basis of July 1, 2016, estimates from the U.S. Census Bureau. The resulting rate approximates the proportion of women of reproductive age who used ART in each reporting area. This proxy measure of ART use is only an approximation because certain women who use ART fall outside the age range of 15–44 years (approximately 9% of cycles performed in 2016) and certain women might have had more than one procedure during the reporting period.

A live-birth delivery was defined as a birth of one or more live-born infants. A singleton live-birth delivery was defined as a birth of only one infant who was born live. A multiple live-birth delivery was defined as a birth of two or more infants, at least one of whom was born live. Low birthweight was defined as <2,500 g, moderate low birthweight as 1,500–2,499 g, and very low birthweight as <1,500 g. Gestational age for births among women who did not undergo ART procedures was calculated using obstetric estimate of gestation at delivery (*26*). For births to women who underwent fresh ART procedures, gestational age was calculated by subtracting the date of egg retrieval from the birth date and adding 14 days. For births to women who underwent frozen embryo cycles or fresh ART procedures for which the date of retrieval was not available, gestational age was calculated by subtracting the date of embryo transfer from the birth date and adding 17 days (to account for an average of 3 days in embryo culture). Preterm delivery was defined as gestational age <37 weeks, late preterm 34–36 weeks, early preterm <34 weeks, and very preterm <32 weeks (*20*).

Elective single-embryo transfer is a procedure in which one embryo, selected from more than one available embryo, is placed in the uterus with one or more embryos cryopreserved. Fresh transfer procedures in which only one embryo was available for transfer and no embryos were cryopreserved are considered single-embryo transfer but not considered eSET. The rate of eSET was calculated by dividing the total number of eSET procedures by the sum of the total number of eSET procedures plus the total number of transfer procedures in which more than one embryo was transferred. The average number of embryos transferred by age group (<35 years, 35–37 years, and >37 years) was calculated by dividing the total number of embryos transferred by the total number of embryo-transfer procedures performed among that age group. In this report, the percentage of eSET procedures and the average number of embryos transferred were calculated only for patients who used fresh embryos from their own fresh eggs, in which at least one embryo was transferred.

The contribution of ART to all infants born in a particular reporting area was used as a second measure of ART use. The contribution of ART to adverse birth outcomes (e.g., preterm, low birthweight, or small for gestational age [SGA] infants) was calculated by dividing the total number of outcomes among ART-conceived infants by the total number of outcomes among all infants born.

The percentage of infants (ART conceived and all infants) born in a reporting area for each plurality group (singleton, multiple, twin, and triplet and higher-order birth) was calculated by dividing the number of infants (ART conceived and all infants) in each plurality group by the total number of infants born (ART conceived and all infants). The percentage of infants with low birthweight and preterm delivery also was calculated for each plurality group (singleton, twin, and triplet and higher-order births) for both ART-conceived infants and all infants by dividing the number of low birthweight or preterm infants in each plurality group by the total number of infants in that plurality group.

In addition, new in 2016, the proportion of infants who were small for gestational age (i.e., born at <10th percentile of birthweight for gestational age) was calculated using gestational age and birthweight information (*27*). The percentage of SGA infants was calculated for births that occurred at <37 weeks (preterm), 37–41 weeks (full term), and 22–44 weeks (all births) by dividing the number of SGA infants in each gestational age category by the total number of singleton infants in that gestational age category for ART-conceived and all infants, respectively.

Infants born in a reporting area during any given year include those who were conceived naturally and those who resulted from ART and other infertility treatments. To assess the proportion of ART births among overall U.S. births in 2016, ART births were aggregated from two reporting years: 1) infants conceived with ART procedures performed in 2015 and born in 2016 (59.9% of the live-birth deliveries reported to NASS for 2016) and 2) infants conceived with ART procedures performed in 2016 and born in 2016 (40.1% of the live-birth deliveries reported to NASS for 2016).

## Results

### Overview of Fertility Clinics

**FIGURE 1:**
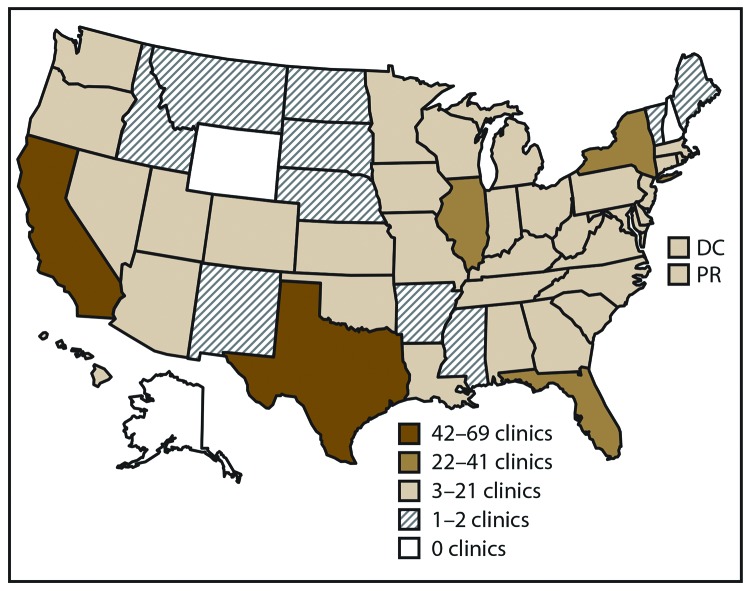
Location and number* of assisted reproductive technology clinics, distributed by quartiles — United States and Puerto Rico, 2016 **Abbreviations:** DC = District of Columbia; PR = Puerto Rico. * In 2016, of the 502 clinics in the United States, 463 (92.2%) submitted data.

In 2016, a total of 502 fertility clinics in the United States performed ART procedures and 463 (92.2%) provided data to CDC, with the majority located in or near major cities (*1*) (Figure 1). The number of fertility clinics performing ART procedures varied by reporting area. The reporting areas with the largest numbers of fertility clinics providing data were California (69), Texas (43), and New York (37).

### Number and Type of ART Procedures

The number, type, and outcome of ART procedures performed are provided according to patient’s residence for all 52 reporting areas (Table 1). Residency data were missing for approximately 0.4% of procedures performed and in this case, the patient’s residence was assigned as the location where the ART procedure was performed. In addition, residency data also were missing for 0.2% of live-birth deliveries; however, these ART procedures were included in the totals. In 2016, approximately 16.6% of ART procedures were conducted in reporting areas other than the patient’s state of residence. Non-U.S. residents accounted for approximately 3.0% of ART procedures, 3.5% of ART live-birth deliveries, and 3.6% of ART-conceived infants born.

**TABLE 1:** Number* and outcomes of assisted reproductive technology procedures with the intent to transfer at least one embryo, by female patient’s reporting area of residence^†^ at time of treatment — United States and Puerto Rico, 2016

Patient’s reporting area of residence	No. of ART clinics^§^	No. of ART procedures performed	No. of ART embryo-transfer procedures^¶^	No. of ART pregnancies	No. of ART live-birth deliveries	No. of ART singleton live-birth deliveries	No. of ART multiple live-birth deliveries	No. of ART live-born infants	ART procedures per 1 million women aged 15–44 yrs**
Alabama	5	1,002	756	364	304	241	63	370	1,051.6
Alaska	0	198	156	77	66	57	9	74	1,343.5
Arizona	15	3,047	2,487	1,384	1,140	859	281	1,425	2,290.3
Arkansas	1	577	453	233	190	150	40	231	1,000.4
California	69	24,030	18,971	10,561	8,638	7,407	1,231	9,885	2,980.5
Colorado	8	2,346	2,044	1,331	1,123	947	176	1,304	2,088.8
Connecticut	8	3,617	2,728	1,511	1,248	1,027	221	1,474	5,364.6
Delaware	2	770	565	299	223	210	13	236	4,284.5
District of Columbia	3	1,352	1,006	475	382	360	22	405	7,371.3
Florida	27	8,714	6,580	3,300	2,695	2,180	515	3,213	2,310.6
Georgia	8	4,270	3,528	1,934	1,537	1,299	238	1,780	2,005.9
Hawaii	5	1,160	807	444	344	260	84	432	4,341.2
Idaho	1	576	470	280	222	161	61	284	1,788.3
Illinois^††^	27	12,816	9,842	4,841	3,887	3,286	601	4,496	5,026.6
Indiana	9	2,282	1,779	859	715	568	147	864	1,764.6
Iowa	2	1,362	1,104	693	587	509	78	666	2,311.1
Kansas	4	1,056	806	476	386	318	68	454	1,890.3
Kentucky	6	1,374	1,153	570	470	372	98	570	1,619.4
Louisiana	6	1,724	1,150	630	511	390	121	635	1,833.1
Maine	1	491	422	220	177	146	31	208	2,122.4
Maryland	7	6,545	5,127	2,494	1,929	1,734	195	2,129	5,484.2
Massachusetts^††^	8	10,095	8,283	3,921	3,220	2,908	312	3,537	7,352.1
Michigan	13	4,213	3,349	1,773	1,439	1,066	373	1,816	2,248.2
Minnesota	5	2,919	2,506	1,445	1,190	964	226	1,419	2,767.6
Mississippi	2	569	457	244	205	169	36	243	955.4
Missouri	9	2,523	2,070	1,065	906	701	205	1,117	2,154.0
Montana	1	335	262	145	123	94	29	152	1,782.1
Nebraska	2	811	620	345	295	235	60	356	2,202.8
Nevada	5	1,321	1,084	654	524	420	104	627	2,279.0
New Hampshire	0	819	674	304	253	223	30	285	3,400.1
New Jersey^††^	20	11,704	8,369	4,643	3,827	3,356	471	4,305	6,855.4
New Mexico	2	291	274	138	106	84	22	130	738.6
New York	37	23,665	17,935	8,236	6,448	5,547	901	7,367	5,914.7
North Carolina	11	4,307	3,331	1,888	1,537	1,230	307	1,849	2,150.7
North Dakota	1	310	253	129	110	79	31	142	2,104.2
Ohio	13	4,538	3,646	1,907	1,586	1,245	341	1,932	2,065.2
Oklahoma	3	984	796	399	338	261	77	416	1,280.5
Oregon	3	1,314	1,112	698	593	453	140	738	1,645.8
Pennsylvania	16	7,635	5,796	2,761	2,266	1,958	308	2,580	3,203.3
Puerto Rico	3	261	228	103	57	42	15	74	385.2
Rhode Island^††^	1	815	697	297	234	194	40	274	3,908.7
South Carolina	4	1,748	1,329	753	594	475	119	715	1,825.0
South Dakota	1	288	235	119	106	88	18	125	1,821.9
Tennessee	10	1,760	1,398	766	643	537	106	753	1,350.8
Texas	43	14,362	11,383	6,302	5,178	4,193	985	6,178	2,473.4
Utah	3	2,073	1,766	1,011	829	665	164	997	3,128.2
Vermont	2	317	241	115	100	82	18	117	2,771.2
Virginia	9	5,668	4,488	2,269	1,807	1,601	206	2,015	3,381.5
Washington	12	4,129	3,166	1,724	1,437	1,260	177	1,621	2,872.4
West Virginia	3	357	307	147	122	101	21	144	1,089.3
Wisconsin	7	2,093	1,713	904	762	609	153	917	1,935.1
Wyoming	0	162	137	80	64	51	13	76	1,472.9
Nonresident	—	6,011	4,600	2,710	2,291	1,846	445	2,740	—^§§^
**Total**	**463**	**197,706**	**154,439**	**80,971**	**65,964**	**55,218**	**10,746**	**76,892**	**3,075.2**

**Abbreviation:** ART = assisted reproductive technology.

* Excludes 65,840 cycles with the intent to freeze all eggs or embryos and 31 procedures performed in territories not included in this report.

^†^ In cases of missing residency data (0.4%), the patient’s residence was assigned as the location where the ART procedure was performed.

^§^ The ART procedures and outcomes by patient’s reporting area of residence do not necessarily reflect the procedures and outcomes of the ART clinics within the reporting area because some patients seek treatment at a clinic in a location other than their area of residence.

^¶^ Embryo-transfer procedures include all procedures performed in which an attempt was made to transfer at least one embryo.

** On the basis of U.S. Census Bureau estimates. **Source:** US Census Bureau. Annual estimates of the resident population for selected age groups by sex for the United States, states, counties, and Puerto Rico Commonwealth and municipios: April 1, 2010 to July 1, 2016. Washington, DC: US Census Bureau, Population Division; 2016. https://factfinder.census.gov/faces/tableservices/jsf/pages/productview.xhtml?pid=PEP_2016_PEPAGESEX&prodType=table.

^††^ State with comprehensive insurance mandate requiring insurers to cover the costs associated with diagnosis and treatment of infertility inclusive of ART services for at least four oocyte retrievals.

^§§^ Non-U.S. residents were excluded from rate because the appropriate denominators were not available.

**FIGURE 2:**
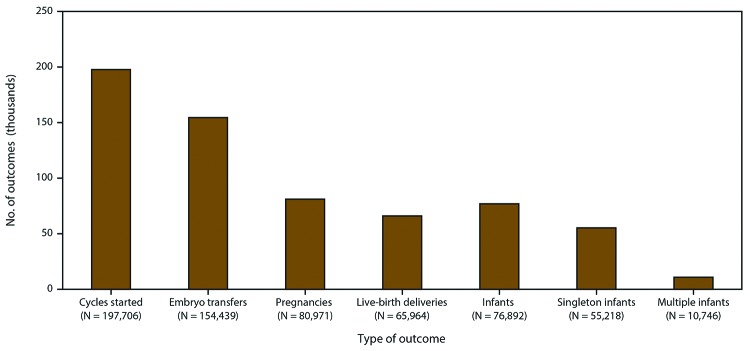
Number of outcomes of assisted reproductive technology procedures* with the intent to transfer at least one embryo, by type of outcome — United States and Puerto Rico, 2016 * A total of 263,577 assisted reproductive technology procedures were reported to CDC.

In 2016, a total of 263,577 ART procedures were reported to CDC (*1*). Included in this report are data for 197,706 ART procedures performed (range: 162 in Wyoming to 24,030 in California) in the United States (including Puerto Rico) with the intent to transfer at least one embryo (Table 1) (Figure 2). Excluded are 65,840 egg or embryo-freezing and embryo-banking procedures that did not result in an embryo transfer as well as 31 procedures that were performed in territories not included in this report. Of 197,706 procedures performed in the reporting areas, 154,439 (78.1%) progressed to embryo transfer. Of 154,439 ART procedures that progressed to the embryo-transfer stage, 80,971 (52.4%) resulted in a pregnancy and 65,964 (42.7%) in a live-birth delivery (range: 57 in Puerto Rico to 8,638 in California). The 65,964 live-birth deliveries included 55,218 singleton live-birth deliveries (83.7%) and 10,746 multiple live-birth deliveries (16.3%) and resulted in 76,892 live-born infants (range: 74 in Alaska to 9,885 in California).

Six reporting areas with the largest numbers of ART procedures (California, Illinois, Massachusetts, New Jersey, New York, and Texas) accounted for approximately half (48.9%; 96,672 of 197,706) of all ART procedures, 48.4% (74,783 of 154,439) of all embryo-transfer procedures, 46.5% (35,768 of 76,892) of all ART-conceived infants born, and 41.9% (4,501 of 10,746) of all ART-conceived multiple live-birth deliveries in the United States (Table 1). However, these six reporting areas accounted for only 36.5% of all U.S. births (*24*).

**FIGURE 3:**
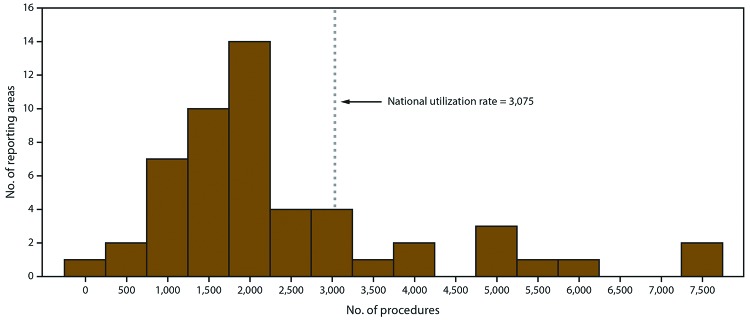
Number of reporting areas,* by number of assisted reproductive technology procedures performed^†^ with the intent to transfer at least one embryo among women of reproductive age (15–44 years)^§^ — United States and Puerto Rico, 2016 * Total number of reporting areas: 52. ^†^ Total number of procedures: 197,706. ^§^ Per 1 million women aged 15–44 years.

The number of ART procedures per 1 million women of reproductive age (15–44 years) ranged from 385 in Puerto Rico to 7,371 in the District of Columbia, with an overall national rate of 3,075 (Table 1) (Figure 3). Fourteen reporting areas (Connecticut, Delaware, Hawaii, Illinois, Maryland, Massachusetts, New Hampshire, New Jersey, New York, Pennsylvania, Rhode Island, Utah, Virginia, and the District of Columbia) had ART use rates higher than the national rate. Of these reporting areas, the District of Columbia (7,371), Massachusetts (7,352), and New Jersey 6,855) had rates exceeding twice the national rate, whereas Connecticut (5,365), Delaware (4,285), Hawaii (4,341), Illinois (5,027), Maryland (5,484), and New York (5,915) had rates exceeding 1.5 times the national rate. The three reporting areas with the lowest ART use rates were Puerto Rico (385), New Mexico (739), and Mississippi (955).

### Number of Embryos Transferred

The number of embryo-transfer procedures performed, the average number of embryos transferred per procedure, and the percentage of eSET procedures performed among women who used fresh embryos from their own fresh eggs are provided by reporting area and age group (Table 2). Overall, 24,876 embryo-transfer procedures were performed among women aged <35 years, 11,688 among women aged 35–37 years, and 16,131 among women aged >37 years. Nationally, on average, 1.5 embryos were transferred per procedure among women aged <35 years, 1.7 embryos among women aged 35–37 years, and 2.2 embryos among women aged >37 years. The national eSET rate was 42.7% among women aged <35 years (range: 8.3% in North Dakota to 83.9% in Delaware), 25.2% among women aged 35–37 years (range: 2.6% in Puerto Rico to 50.6% in Massachusetts), and 6.7% among women aged >37 years (range: 0% in multiple reporting areas to 31.3% in Delaware).

**TABLE 2:** Number of assisted reproductive technology embryo-transfer procedures with the intent to transfer at least one embryo* among patients who used fresh embryos from their own fresh eggs, by female patient’s age group and reporting area of residence^†^ at time of treatment — United States and Puerto Rico, 2016

Patient’s reporting area of residence	<35 yrs			35–37 yrs			>37 yrs		
	No. of embryo-transfer procedures	Average no. of embryos transferred	eSET^§^ (%)	No. of embryo-transfer procedures	Average no. of embryos transferred	eSET (%)	No. of embryo-transfer procedures	Average no. of embryos transferred	eSET (%)
Alabama	176	1.7	26.9	48	1.8	7.7	51	2.2	0.0
Alaska	30	1.6	37.0	18	—^¶^	—^¶^	11	—^¶^	—^¶^
Arizona	268	1.6	33.7	141	1.9	15.8	133	2.4	0.9
Arkansas	126	1.7	23.4	43	1.9	8.8	22	2.0	5.9
California	1,923	1.5	42.8	1,246	1.7	31.1	1,949	2.3	7.9
Colorado	131	1.7	27.5	61	1.8	11.5	32	1.9	8.0
Connecticut	562	1.4	52.1	282	1.7	26.6	370	2.1	8.6
Delaware	33	1.2	83.9	19	—^¶^	—^¶^	22	1.7	31.3
District of Columbia	110	1.3	65.4	81	1.4	48.2	198	1.8	9.7
Florida	939	1.6	37.9	463	1.8	16.5	680	2.2	3.0
Georgia	567	1.5	48.1	224	1.7	19.8	285	2.2	6.7
Hawaii	69	1.8	20.6	37	2.2	2.9	89	2.5	2.7
Idaho	104	1.6	34.4	33	1.8	14.3	17	—^¶^	—^¶^
Illinois**	1,957	1.6	39.1	954	1.7	22.2	1,304	2.1	4.8
Indiana	478	1.7	18.3	142	1.9	7.6	172	2.2	4.3
Iowa	266	1.3	62.7	80	1.5	49.3	72	1.6	23.5
Kansas	120	1.5	43.4	39	1.8	11.4	27	2.1	4.8
Kentucky	342	1.6	35.7	138	1.9	7.0	88	2.4	0.0
Louisiana	164	1.7	32.4	58	1.7	11.4	41	2.2	3.1
Maine	99	1.3	70.6	41	1.5	41.2	64	2.1	7.4
Maryland	1,041	1.3	64.1	526	1.5	43.7	782	2.0	9.4
Massachusetts**	1,545	1.2	73.4	863	1.4	50.6	1,382	2.2	10.6
Michigan	831	1.7	22.1	289	1.9	6.9	349	2.1	3.7
Minnesota	628	1.5	45.6	241	1.7	22.9	192	2.1	8.2
Mississippi	60	1.4	52.1	29	1.9	15.4	33	2.1	3.8
Missouri	426	1.7	22.2	174	1.8	14.0	133	2.3	1.8
Montana	44	1.4	53.7	16	—^¶^	—^¶^	28	2.2	0.0
Nebraska	141	1.5	35.4	30	1.9	4.0	27	2.3	0.0
Nevada	149	1.7	23.1	37	1.8	17.1	49	1.6	21.2
New Hampshire	164	1.3	63.3	65	1.5	39.2	77	2.1	4.9
New Jersey**	1,012	1.4	50.0	550	1.7	24.3	857	2.0	7.6
New Mexico	33	1.8	10.0	15	—^¶^	—^¶^	12	—^¶^	—^¶^
New York	2,544	1.6	40.9	1,393	1.8	23.7	2,884	2.3	5.3
North Carolina	564	1.5	46.9	258	1.8	15.2	259	2.2	2.8
North Dakota	58	1.8	8.3	18	—^¶^	—^¶^	22	2.0	5.3
Ohio	982	1.6	34.0	349	1.8	14.0	298	2.3	2.7
Oklahoma	262	1.7	21.8	79	2.0	7.1	61	2.1	1.9
Oregon	148	1.7	21.1	60	1.7	19.1	65	1.9	4.1
Pennsylvania	1,111	1.5	47.8	468	1.7	29.1	532	2.1	8.2
Puerto Rico	66	1.9	10.0	40	2.3	2.6	64	2.2	0.0
Rhode Island**	167	1.4	60.0	85	1.7	32.9	139	2.5	3.2
South Carolina	213	1.7	26.4	83	2.0	6.4	77	2.1	7.4
South Dakota	74	1.8	20.6	19	—^¶^	—^¶^	7	—^¶^	—^¶^
Tennessee	197	1.6	36.9	75	1.8	13.6	77	2.3	3.1
Texas	1,466	1.6	33.5	663	1.8	14.0	781	2.0	3.1
Utah	524	1.5	41.8	148	1.7	23.3	90	2.0	5.3
Vermont	60	1.5	34.1	37	1.8	14.3	52	1.8	16.7
Virginia	732	1.3	59.4	377	1.4	45.2	568	1.8	13.7
Washington	453	1.5	50.6	233	1.7	27.6	270	1.9	7.2
West Virginia	87	1.7	24.4	31	1.9	11.5	18	—^¶^	—^¶^
Wisconsin	334	1.6	38.1	111	1.7	27.4	96	2.1	3.8
Wyoming	29	1.6	37.9	12	—^¶^	—^¶^	7	—^¶^	—^¶^
Nonresident	267	1.6	34.3	146	1.7	26.3	216	2.0	11.4
**Total**	**24,876**	**1.5**	**42.7**	**11,668**	**1.7**	**25.2**	**16,131**	**2.2**	**6.7**

**Abbreviation:** eSET = elective single-embryo transfer.

* Includes all procedures in which at least one embryo was transferred.

^†^ In cases of missing residency data (0.4%), the patient’s residence was assigned as the location where the assisted reproductive technology procedure was performed.

^§^ A procedure in which one embryo, selected from a larger number of available embryos, is placed in the uterus. A cycle in which only one embryo is available is not defined as eSET.

^¶^ Estimates on the basis of N = <20 in the denominator have been suppressed because such rates are considered unstable.

** State with comprehensive insurance mandate requiring insurers to cover the costs associated with diagnosis and treatment of infertility inclusive of ART services for at least four oocyte retrievals.

### Singleton and Multiple-Birth Infants

In 2016, among 3,974,132 infants born in the United States and Puerto Rico (*21*), 70,600 (1.8%) were conceived with ART procedures performed in 2015 and 2016 (Table 3). California, Texas, and New York had the highest total numbers of all infants born (488,827, 398,047, and 234,283, respectively) and ART-conceived infants born (9,305, 6,113, and 6,981, respectively). The percentage of ART-conceived infants born among all infants born was highest in Massachusetts (4.7%) followed by Connecticut and New Jersey (3.9% each).

**TABLE 3:** Number, proportion, and percentage of infants born with use of assisted reproductive technology, by female patient’s reporting area of residence* at time of treatment — United States and Puerto Rico, 2016^†^

Patient’s reporting area of residence	Total no. of infants born^§,¶^	No. of ART infants born	Proportion of ART infants among all infants (%)	Singleton infants among ART infants	Singleton infants among all infants^¶^	Proportion of ART singleton infants among all singleton infants (%)
				No. (%)	No. (%)	
Alabama	59,151	445	0.8	256 (57.5)	56,878 (96.2)	0.5
Alaska	11,209	83	0.7	46 (55.4)	10,832 (96.6)	0.4
Arizona	84,520	1,154	1.4	697 (60.4)	81,864 (96.9)	0.9
Arkansas	38,274	220	0.6	134 (60.9)	37,154 (97.1)	0.4
California	488,827	9,305	1.9	6,685 (71.8)	473,330 (96.8)	1.4
Colorado	66,613	1,229	1.8	835 (67.9)	64,499 (96.8)	1.3
Connecticut	36,015	1,392	3.9	906 (65.1)	34,453 (95.7)	2.6
Delaware	10,992	273	2.5	235 (86.1)	10,667 (97.0)	2.2
District of Columbia	9,858	350	3.6	293 (83.7)	9,470 (96.1)	3.1
Florida	225,022	3,065	1.4	2,008 (65.5)	217,688 (96.7)	0.9
Georgia	130,042	1,569	1.2	1,153 (73.5)	125,619 (96.6)	0.9
Hawaii	18,059	407	2.3	243 (59.7)	17,435 (96.5)	1.4
Idaho	22,482	291	1.3	172 (59.1)	21,739 (96.7)	0.8
Illinois**	154,445	4,454	2.9	3,048 (68.4)	148,504 (96.2)	2.1
Indiana	83,091	862	1.0	543 (63.0)	80,234 (96.6)	0.7
Iowa	39,403	711	1.8	498 (70.0)	38,044 (96.6)	1.3
Kansas	38,053	438	1.2	307 (70.1)	36,905 (97.0)	0.8
Kentucky	55,449	536	1.0	351 (65.5)	53,436 (96.4)	0.7
Louisiana	63,178	538	0.9	342 (63.6)	60,929 (96.4)	0.6
Maine	12,705	194	1.5	149 (76.8)	12,306 (96.9)	1.2
Maryland	73,136	2,036	2.8	1,605 (78.8)	70,585 (96.5)	2.3
Massachusetts**	71,317	3,356	4.7	2,659 (79.2)	68,656 (96.3)	3.9
Michigan	113,315	1,781	1.6	964 (54.1)	108,940 (96.1)	0.9
Minnesota	69,749	1,239	1.8	776 (62.6)	67,296 (96.5)	1.2
Mississippi	37,928	234	0.6	160 (68.4)	36,661 (96.7)	0.4
Missouri	74,705	1,027	1.4	634 (61.7)	72,018 (96.4)	0.9
Montana	12,282	146	1.2	80 (54.8)	11,897 (96.9)	0.7
Nebraska	26,589	319	1.2	207 (64.9)	25,643 (96.4)	0.8
Nevada	36,260	603	1.7	403 (66.8)	35,123 (96.9)	1.1
New Hampshire	12,267	270	2.2	205 (75.9)	11,895 (97.0)	1.7
New Jersey**	102,647	4,002	3.9	2,961 (74.0)	98,641 (96.1)	3.0
New Mexico	24,692	98	0.4	59 (60.2)	24,035 (97.3)	0.2
New York	234,283	6,981	3.0	4,991 (71.5)	225,522 (96.3)	2.2
North Carolina	120,779	1,682	1.4	1,079 (64.1)	116,530 (96.5)	0.9
North Dakota	11,383	140	1.2	63 (45.0)	11,004 (96.7)	0.6
Ohio	138,085	1,962	1.4	1,167 (59.5)	132,936 (96.3)	0.9
Oklahoma	52,592	398	0.8	230 (57.8)	50,914 (96.8)	0.5
Oregon	45,535	644	1.4	395 (61.3)	43,986 (96.6)	0.9
Pennsylvania	139,409	2,373	1.7	1,747 (73.6)	134,533 (96.5)	1.3
Puerto Rico	28,257	81	0.3	46 (56.8)	27,687 (98.0)	0.2
Rhode Island**	10,798	229	2.1	160 (69.9)	10,389 (96.2)	1.5
South Carolina	57,342	729	1.3	415 (56.9)	55,202 (96.3)	0.8
South Dakota	12,275	147	1.2	73 (49.7)	11,825 (96.3)	0.6
Tennessee	80,807	707	0.9	457 (64.6)	78,075 (96.6)	0.6
Texas	398,047	6,113	1.5	3,938 (64.4)	385,145 (96.8)	1.0
Utah	50,464	1,021	2.0	609 (59.6)	48,547 (96.2)	1.3
Vermont	5,756	106	1.8	72 (67.9)	5,568 (96.7)	1.3
Virginia	102,460	2,097	2.0	1,529 (72.9)	98,834 (96.5)	1.5
Washington	90,505	1,511	1.7	1,115 (73.8)	87,792 (97.0)	1.3
West Virginia	19,079	130	0.7	81 (62.3)	18,491 (96.9)	0.4
Wisconsin	66,615	862	1.3	545 (63.2)	64,318 (96.6)	0.8
Wyoming	7,386	60	0.8	41 (68.3)	7,162 (97.0)	0.6
**Total**	**3,974,132**	**70,600**	**1.8**	**48,367 (68.5)**	**3,837,836 (96.6)**	**1.3**

**Abbreviation:** ART = assisted reproductive technology.

* In cases of missing residency data (0.4%), the patient’s residence was assigned as the location where the ART procedure was performed.

^†^ Includes infants conceived from ART procedures performed in 2015 and born in 2016 and infants conceived from ART procedures performed in 2016 and born in 2016. Total ART births exclude births to nonresidents.

^§^ U.S. births include births to nonresidents**. Source:** Martin JA, Hamilton BE, Osterman MJ, Driscoll AK, Mathews TJ. Births: final data for 2016. Natl Vital Stat Rep 2018;67:1–55.

^¶^ U.S. births include births to nonresidents**.** **Source:** CDC Wonder [Internet]. Natality public use data 2007–2016. Atlanta, GA: US Department of Health and Human Services, CDC; 2018.

** State with comprehensive insurance mandate requiring insurers to cover the costs associated with diagnosis and treatment of infertility inclusive of ART services for at least four oocyte retrievals.

Nationally, 31.5% of ART-conceived infants were born in multiple-birth deliveries (range: 13.9% in Delaware to 55.0% in North Dakota), compared with 3.4% of all infants (range: 2.0% in Puerto Rico to 4.3% in Connecticut) (Table 4). ART-conceived twins accounted for approximately 96.5% (21,455 of 22,233) of all ART-conceived infants born in multiple deliveries. ART-conceived multiple-birth infants contributed to 16.4% of all multiple-birth infants (range: 5.8% in Mississippi to 31.1% in Connecticut). Approximately 30.4% of all ART-conceived infants were twins compared with 3.3% of all infants. ART-conceived twins contributed to 16.2% of all twins. Of ART-conceived infants, 1.1% were triplets and higher-order multiples compared with 0.1% among all infants. ART-conceived triplets and higher-order infants contributed to 19.4% of all triplets and higher-order infants.

**TABLE 4:** Number, percentage, and proportion of multiple-birth infants, twins, and triplets and higher-order infants born with use of assisted reproductive technology procedures, by female patient’s reporting area of residence* at time of treatment — United States and Puerto Rico, 2016^†^

Patient’s reporting area of residence	Multiple-birth infants among ART infants^§^	Multiple-birth infants among all infants^¶^	Proportion of ART multiple-birth infants among all multiple-birth infants (%)	Twin infants among ART infants^§^	Twin infants among all infants^¶^	Proportion of ART twin infants among all twin infants (%)	Triplets and higher-order infants among ART infants^§^	Triplets and higher-order infants among all infants^¶^	Proportion of ART triplets and higher-order infants among all triplets and higher-order infants (%)
	No. (%)	No. (%)		No. (%)	No. (%)		No. (%)	No. (%)	
Alabama	189 (42.5)	2,273 (3.8)	8.3	174 (39.1)	2,182 (3.7)	8.0	15 (3.4)	91 (0.2)	16.5
Alaska	37 (44.6)	— (—)**	—**	37 (44.6)	374 (3.3)	9.9	0 (0.0)	— (—)**	—**
Arizona	457 (39.6)	2,656 (3.1)	17.2	429 (37.2)	2,583 (3.1)	16.6	28 (2.4)	73 (0.1)	38.4
Arkansas	86 (39.1)	1,120 (2.9)	7.7	86 (39.1)	1,093 (2.9)	7.9	0 (0.0)	27 (0.1)	0.0
California	2,620 (28.2)	15,497 (3.2)	16.9	2,535 (27.2)	15,068 (3.1)	16.8	85 (0.9)	429 (0.1)	19.8
Colorado	394 (32.1)	2,114 (3.2)	18.6	381 (31.0)	2,046 (3.1)	18.6	13 (1.1)	68 (0.1)	19.1
Connecticut	486 (34.9)	1,562 (4.3)	31.1	462 (33.2)	1,511 (4.2)	30.6	24 (1.7)	51 (0.1)	47.1
Delaware	38 (13.9)	— (—)**	—**	38 (13.9)	325 (3.0)	11.7	0 (0.0)	— (—)**	—**^,††^
District of Columbia	57 (16.3)	— (—)**	—**	— (—)**	388 (3.9)	—**	— (—)**	— (—)**	—**^,††^
Florida	1,057 (34.5)	7,334 (3.3)	14.4	1,015 (33.1)	7,162 (3.2)	14.2	42 (1.4)	172 (0.1)	24.4
Georgia	416 (26.5)	4,423 (3.4)	9.4	404 (25.7)	4,271 (3.3)	9.5	12 (0.8)	152 (0.1)	7.9
Hawaii	164 (40.3)	624 (3.5)	26.3	155 (38.1)	590 (3.3)	26.3	9 (2.2)	34 (0.2)	26.5
Idaho	119 (40.9)	743 (3.3)	16.0	113 (38.8)	715 (3.2)	15.8	6 (2.1)	28 (0.1)	21.4
Illinois	1,406 (31.6)	5,941 (3.8)	23.7	1,357 (30.5)	5,772 (3.7)	23.5	49 (1.1)	169 (0.1)	29.0
Indiana	319 (37.0)	2,857 (3.4)	11.2	304 (35.3)	2,744 (3.3)	11.1	15 (1.7)	113 (0.1)	13.3
Iowa	213 (30.0)	1,359 (3.4)	15.7	204 (28.7)	1,310 (3.3)	15.6	9 (1.3)	49 (0.1)	18.4
Kansas	131 (29.9)	1,148 (3.0)	11.4	125 (28.5)	1,111 (2.9)	11.3	6 (1.4)	37 (0.1)	16.2
Kentucky	185 (34.5)	2,013 (3.6)	9.2	170 (31.7)	1,938 (3.5)	8.8	15 (2.8)	75 (0.1)	20.0
Louisiana	196 (36.4)	2,249 (3.6)	8.7	190 (35.3)	2,157 (3.4)	8.8	6 (1.1)	92 (0.1)	6.5
Maine	45 (23.2)	399 (3.1)	11.3	— (—)**	384 (3.0)	—**	— (—)**	15 (0.1)	—**^,††^
Maryland	431 (21.2)	2,551 (3.5)	16.9	414 (20.3)	2,475 (3.4)	16.7	17 (0.8)	76 (0.1)	22.4
Massachusetts	697 (20.8)	2,661 (3.7)	26.2	685 (20.4)	2,571 (3.6)	26.6	12 (0.4)	90 (0.1)	13.3
Michigan	817 (45.9)	4,375 (3.9)	18.7	790 (44.4)	4,239 (3.7)	18.6	27 (1.5)	136 (0.1)	19.9
Minnesota	463 (37.4)	2,453 (3.5)	18.9	454 (36.6)	2,401 (3.4)	18.9	9 (0.7)	52 (0.1)	17.3
Mississippi	74 (31.6)	1,267 (3.3)	5.8	— (—)**	1,231 (3.2)	—**	— (—)**	36 (0.1)	—**
Missouri	393 (38.3)	2,687 (3.6)	14.6	375 (36.5)	2,594 (3.5)	14.5	18 (1.8)	93 (0.1)	19.4
Montana	66 (45.2)	— (—)**	—**	66 (45.2)	379 (3.1)	17.4	0 (0.0)	— (—)**	—**^,††^
Nebraska	112 (35.1)	946 (3.6)	11.8	112 (35.1)	898 (3.4)	12.5	0 (0.0)	48 (0.2)	0.0
Nevada	200 (33.2)	1,137 (3.1)	17.6	— (—)**	1,115 (3.1)	—**	— (—)**	22 (0.1)	—**
New Hampshire	65 (24.1)	372 (3.0)	17.5	— (—)**	351 (2.9)	—**	— (—)**	21 (0.2)	—**
New Jersey	1,041 (26.0)	4,006 (3.9)	26.0	1,018 (25.4)	3,900 (3.8)	26.1	23 (0.6)	106 (0.1)	21.7
New Mexico	39 (39.8)	657 (2.7)	5.9	— (—)**	642 (2.6)	—**	— (—)**	15 (0.1)	—**^,††^
New York	1,990 (28.5)	8,761 (3.7)	22.7	1,927 (27.6)	8,539 (3.6)	22.6	63 (0.9)	222 (0.1)	28.4
North Carolina	603 (35.9)	4,249 (3.5)	14.2	576 (34.2)	4,126 (3.4)	14.0	27 (1.6)	123 (0.1)	22.0
North Dakota	77 (55.0)	379 (3.3)	20.3	— (—)**	364 (3.2)	—**	— (—)**	15 (0.1)	—**^,††^
Ohio	795 (40.5)	5,149 (3.7)	15.4	756 (38.5)	4,975 (3.6)	15.2	39 (2.0)	174 (0.1)	22.4
Oklahoma	168 (42.2)	1,678 (3.2)	10.0	159 (39.9)	1,641 (3.1)	9.7	9 (2.3)	37 (0.1)	24.3
Oregon	249 (38.7)	1,549 (3.4)	16.1	240 (37.3)	1,501 (3.3)	16.0	9 (1.4)	48 (0.1)	18.8
Pennsylvania	626 (26.4)	4,876 (3.5)	12.8	611 (25.7)	4,766 (3.4)	12.8	15 (0.6)	110 (0.1)	13.6
Puerto Rico	35 (43.2)	570 (2.0)	6.1	35 (43.2)	556 (2.0)	6.3	0 (0.0)	14 (0.0)	—^††^
Rhode Island	69 (30.1)	409 (3.8)	16.9	— (—)**	393 (3.6)	—**	— (—)**	16 (0.1)	—**^,††^
South Carolina	314 (43.1)	2,140 (3.7)	14.7	288 (39.5)	2,063 (3.6)	14.0	26 (3.6)	77 (0.1)	33.8
South Dakota	74 (50.3)	450 (3.7)	16.4	62 (42.2)	429 (3.5)	14.5	12 (8.2)	21 (0.2)	57.1
Tennessee	250 (35.4)	2,732 (3.4)	9.2	235 (33.2)	2,641 (3.3)	8.9	15 (2.1)	91 (0.1)	16.5
Texas	2,175 (35.6)	12,902 (3.2)	16.9	2,124 (34.7)	12,505 (3.1)	17.0	51 (0.8)	397 (0.1)	12.8
Utah	412 (40.4)	1,917 (3.8)	21.5	403 (39.5)	1,863 (3.7)	21.6	9 (0.9)	54 (0.1)	16.7
Vermont	34 (32.1)	— (—)**	—**	— (—)**	185 (3.2)	—**	— (—)**	— (—)**	—**^,††^
Virginia	568 (27.1)	3,626 (3.5)	15.7	559 (26.7)	3,534 (3.4)	15.8	9 (0.4)	92 (0.1)	9.8
Washington	396 (26.2)	2,713 (3.0)	14.6	375 (24.8)	2,649 (2.9)	14.2	21 (1.4)	64 (0.1)	32.8
West Virginia	49 (37.7)	588 (3.1)	8.3	— (—)**	565 (3.0)	—**	— (—)**	23 (0.1)	—**
Wisconsin	317 (36.8)	2,297 (3.4)	13.8	— (—)**	2,243 (3.4)	—**	— (—)**	54 (0.1)	—**
Wyoming	19 (31.7)	— (—)**	—**	19 (31.7)	221 (3.0)	8.6	0 (0.0)	— (—)**	—**^,††^
**Total**	**22,233 (31.5)**	**135,740 (3.4)**	**16.4**	**21,455 (30.4)**	**132,279 (3.3)**	**16.2**	**778 (1.1)**	**4,017 (0.1)**	**19.4**

**Abbreviation:** ART = assisted reproductive technology.

* In cases of missing residency data (0.4%), the patient’s residence was assigned as the location where the ART procedure was performed.

^†^ ART totals include infants conceived from ART procedures performed in 2015 and born in 2016 and infants conceived from ART procedures performed in 2016 and born in 2016. Total ART births exclude births to nonresidents.

^§^ Includes only the number of infants live born in a multiple-birth delivery. For example, if three infants were born in a live-birth delivery and one of the three infants was stillborn, the total number of live-born infants would be two. However, the two infants still would be counted as triplets.

^¶^ U.S. births include births to nonresidents. **Source:** CDC Wonder [Internet]. Natality public use data 2007–2016. Atlanta, GA: US Department of Health and Human Services, CDC; 2018.

** To protect confidentiality, cells with values of 1–4 for ART infants and cells with values of 0–9 for all infants are suppressed. Also suppressed are data that can be used to derive suppressed cell values. These values are included in the totals.

^††^ Estimates on the basis of N = <20 in the denominator have been suppressed because such rates are considered unstable.

### Adverse Perinatal Outcomes

Nationally, ART-conceived infants contributed to approximately 5.0% of all infants with low birthweight, 5.1% of all infants with moderate low birthweight, and 5.0% of all infants with very low birthweight (Table 5). In three reporting areas (Connecticut, Massachusetts, and New Jersey), >10% of all infants with low birthweight were conceived with ART. Among ART-conceived infants, 23.6% had low birthweight compared with 8.2% among all infants. Approximately 4.0% of ART-conceived infants had very low birthweight compared with 1.4% among all infants.

**TABLE 5:** Number, percentage, and proportion of infants born with use of assisted reproductive technology,* by low birthweight category and female patient’s reporting area of residence^†^ at time of treatment — United States and Puerto Rico, 2016

Patient’s reporting area of residence	<1,500 g (VLBW)		Proportion of ART VLBW infants among all VLBW infants (%)	1,500–2,499 g (MLBW)			<2,500 g (LBW)		
	ART infants	All infants^§^		ART infants	All infants^§^	Proportion of ART MLBW infants among all MLBW infants (%)	ART infants	All infants^§^	Proportion of ART LBW infants among all LBW infants (%)
	No. (%)	No. (%)		No. (%)	No. (%)		No. (%)	No. (%)	
Alabama	21 (4.7)	1,165 (2.0)	1.8	129 (29.1)	4,931 (8.3)	2.6	150 (33.9)	6,096 (10.3)	2.5
Alaska	6 (7.4)	109 (1.0)	5.5	21 (25.9)	552 (4.9)	3.8	27 (33.3)	661 (5.9)	4.1
Arizona	58 (5.1)	991 (1.2)	5.9	264 (23.0)	5,186 (6.1)	5.1	322 (28.1)	6,177 (7.3)	5.2
Arkansas	14 (6.4)	597 (1.6)	2.3	47 (21.4)	2,764 (7.2)	1.7	61 (27.7)	3,361 (8.8)	1.8
California	302 (3.3)	5,384 (1.1)	5.6	1624 (17.9)	28,092 (5.7)	5.8	1,926 (21.2)	33,476 (6.8)	5.8
Colorado	45 (3.8)	768 (1.2)	5.9	292 (24.5)	5,193 (7.8)	5.6	337 (28.3)	5,961 (8.9)	5.7
Connecticut	46 (3.3)	508 (1.4)	9.1	271 (19.6)	2,305 (6.4)	11.8	317 (22.9)	2,813 (7.8)	11.3
Delaware	10 (3.7)	196 (1.8)	5.1	29 (10.7)	786 (7.2)	3.7	39 (14.4)	982 (8.9)	4.0
District of Columbia	— (—)^¶^	187 (1.9)	—^¶^	— (—)^¶^	811 (8.2)	—^¶^	59 (17.0)	998 (10.1)	5.9
Florida	113 (3.8)	3,405 (1.5)	3.3	628 (20.9)	16,184 (7.2)	3.9	741 (24.7)	19,589 (8.7)	3.8
Georgia	57 (3.7)	2,387 (1.8)	2.4	308 (19.9)	10,317 (7.9)	3.0	365 (23.5)	12,704 (9.8)	2.9
Hawaii	23 (5.9)	265 (1.5)	8.7	100 (25.8)	1,272 (7.0)	7.9	123 (31.7)	1,537 (8.5)	8.0
Idaho	10 (3.4)	243 (1.1)	4.1	65 (22.3)	1,320 (5.9)	4.9	75 (25.8)	1,563 (7.0)	4.8
Illinois	199 (4.5)	2,369 (1.5)	8.4	822 (18.7)	10,618 (6.9)	7.7	1,021 (23.2)	12,987 (8.4)	7.9
Indiana	26 (3.0)	1,219 (1.5)	2.1	181 (21.2)	5,583 (6.7)	3.2	207 (24.2)	6,802 (8.2)	3.0
Iowa	13 (1.8)	469 (1.2)	2.8	113 (16.0)	2,192 (5.6)	5.2	126 (17.9)	2,661 (6.8)	4.7
Kansas	16 (3.7)	423 (1.1)	3.8	78 (18.1)	2,222 (5.8)	3.5	94 (21.8)	2,645 (7.0)	3.6
Kentucky	25 (4.8)	886 (1.6)	2.8	102 (19.5)	4,156 (7.5)	2.5	127 (24.3)	5,042 (9.1)	2.5
Louisiana	32 (6.0)	1,208 (1.9)	2.6	107 (20.2)	5,512 (8.7)	1.9	139 (26.3)	6,720 (10.6)	2.1
Maine	— (—)^¶^	127 (1.0)	—^¶^	— (—)^¶^	770 (6.1)	—^¶^	29 (15.6)	897 (7.1)	3.2
Maryland	80 (3.9)	1,201 (1.6)	6.7	317 (15.6)	5,047 (6.9)	6.3	397 (19.6)	6,248 (8.5)	6.4
Massachusetts	94 (2.9)	833 (1.2)	11.3	491 15.0)	4,497 (6.3)	10.9	585 (17.9)	5,330 (7.5)	11.0
Michigan	86 (4.9)	1,647 (1.5)	5.2	465 (26.5)	8,007 (7.1)	5.8	551 (31.3)	9,654 (8.5)	5.7
Minnesota	32 (2.6)	784 (1.1)	4.1	258 (20.9)	3,786 (5.4)	6.8	290 (23.5)	4,570 (6.6)	6.3
Mississippi	7 (3.0)	803 (2.1)	0.9	55 (23.5)	3,542 (9.3)	1.6	62 (26.5)	4,345 (11.5)	1.4
Missouri	43 (4.4)	1,072 (1.4)	4.0	233 (23.7)	5,401 (7.2)	4.3	276 (28.1)	6,473 (8.7)	4.3
Montana	12 (8.2)	136 (1.1)	8.8	37 (25.3)	830 (6.8)	4.5	49 (33.6)	966 (7.9)	5.1
Nebraska	11 (3.5)	332 (1.2)	3.3	64 (20.2)	1,537 (5.8)	4.2	75 (23.7)	1,869 (7.0)	4.0
Nevada	22 (3.8)	463 (1.3)	4.8	135 (23.6)	2,602 (7.2)	5.2	157 (27.4)	3,065 (8.5)	5.1
New Hampshire	8 (3.0)	121 (1.0)	6.6	40 (14.9)	668 (5.4)	6.0	48 (17.9)	789 (6.4)	6.1
New Jersey	155 (3.9)	1,425 (1.4)	10.9	740 (18.7)	6,847 (6.7)	10.8	895 (22.7)	8,272 (8.1)	10.8
New Mexico	— (—)^¶^	365 (1.5)	—^¶^	— (—)^¶^	1,862 (7.5)	—^¶^	38 (39.6)	2,227 (9.0)	1.7
New York	228 (3.4)	3,120 (1.3)	7.3	1206 (17.9)	15,453 (6.6)	7.8	1,434 (21.3)	18,573 (7.9)	7.7
North Carolina	75 (4.5)	1,976 (1.6)	3.8	350 (21.2)	9,151 (7.6)	3.8	425 (25.7)	11,127 (9.2)	3.8
North Dakota	16 (11.4)	141 (1.2)	11.3	40 (28.6)	611 (5.4)	6.5	56 (40.0)	752 (6.6)	7.4
Ohio	112 (5.8)	2,146 (1.6)	5.2	423 (21.8)	9,835 (7.1)	4.3	535 (27.6)	11,981 (8.7)	4.5
Oklahoma	20 (5.1)	700 (1.3)	2.9	105 (26.6)	3,410 (6.5)	3.1	125 (31.6)	4,110 (7.8)	3.0
Oregon	8 (1.3)	434 (1.0)	1.8	138 (21.9)	2,540 (5.6)	5.4	146 (23.2)	2,974 (6.5)	4.9
Pennsylvania	70 (3.0)	1,963 (1.4)	3.6	390 (16.8)	9,368 (6.7)	4.2	460 (19.8)	11,331 (8.1)	4.1
Puerto Rico	— (—)^¶^	376 (1.3)	—^¶^	— (—)^¶^	2,509 (8.9)	—^¶^	30 (37.5)	2,885 (10.2)	1.0
Rhode Island	9 (4.0)	157 (1.5)	5.7	41 (18.2)	701 (6.5)	5.8	50 (22.2)	858 (7.9)	5.8
South Carolina	41 (5.7)	1,029 (1.8)	4.0	163 (22.8)	4,459 (7.8)	3.7	204 (28.5)	5,488 (9.6)	3.7
South Dakota	6 (4.1)	121 (1.0)	5.0	34 (23.3)	709 (5.8)	4.8	40 (27.4)	830 (6.8)	4.8
Tennessee	42 (6.0)	1,290 (1.6)	3.3	160 (22.9)	6,141 (7.6)	2.6	202 (28.9)	7,431 (9.2)	2.7
Texas	308 (5.1)	5,714 (1.4)	5.4	1353 (22.4)	27,731 (7.0)	4.9	1,661 (27.5)	33,445 (8.4)	5.0
Utah	58 (5.7)	562 (1.1)	10.3	249 (24.7)	3,060 (6.1)	8.1	307 (30.4)	3,622 (7.2)	8.5
Vermont	5 (4.7)	72 (1.3)	6.9	27 (25.5)	322 (5.6)	8.4	32 (30.2)	394 (6.8)	8.1
Virginia	87 (4.2)	1,513 (1.5)	5.8	335 (16.3)	6,750 (6.6)	5.0	422 (20.5)	8,263 (8.1)	5.1
Washington	70 (4.7)	880 (1.0)	8.0	247 (16.4)	4,912 (5.4)	5.0	317 (21.1)	5,792 (6.4)	5.5
West Virginia	— (—)^¶^	270 (1.4)	—^¶^	— (—)^¶^	1,565 (8.2)	—^¶^	31 (24.6)	1,835 (9.6)	1.7
Wisconsin	28 (3.3)	832 (1.2)	3.4	154 (18.0)	4,093 (6.1)	3.8	182 (21.3)	4,925 (7.4)	3.7
Wyoming	— (—)^¶^	102 (1.4)	—^¶^	— (—)^¶^	526 (7.1)	—^¶^	9 (15.3)	628 (8.5)	1.4
**Total**	**2,762 (4.0)**	**55,486 (1.4)**	**5.0**	**13,614 (19.6)**	**269,238 (6.8)**	**5.1**	**16,376 (23.6)**	**324,724 (8.2)**	**5.0**

**Abbreviations:** ART = assisted reproductive technology; LBW = low birthweight; MLBW = moderate low birthweight; VLBW = very low birthweight.

* ART totals include infants conceived from ART procedures performed in 2015 and born in 2016 and infants conceived from ART procedures performed in 2016 and born in 2016. Total ART infants exclude births to nonresidents and include only infants with birthweight data available.

^†^ In cases of missing residency data (0.4%), the patient’s residence was assigned as the location where the ART procedure was performed.

^§^ U.S. births include births to nonresidents. **Source:** CDC Wonder [Internet]. Natality public use data 2007–2016. Atlanta, GA: US Department of Health and Human Services, CDC; 2018.

^¶^ To protect confidentiality, cells with values of 1–4 for ART infants and cells with values of 0–9 for all infants are suppressed. Also suppressed are data that can be used to derive suppressed cell values. These values are included in the totals.

Nationally, ART contributed to approximately 5.5% of all infants born very preterm, 6.0% early preterm, 5.1% late preterm, and 5.3% preterm (Table 6). In Connecticut, Massachusetts, and New Jersey, the contribution of ART to preterm infants exceeded >10% in all categories of preterm birth. Among ART-conceived infants, rates for preterm birth were 5% very preterm, 9.5% early preterm, 20.4% late preterm, and 29.9% preterm. Corresponding rates of preterm birth among all infants born were 1.6% (<32 weeks), 2.8% (<34 weeks), 7.1% (34–36 weeks), and 9.9% (<37 weeks). Infants born at gestational weeks 34–36 (late preterm births) accounted for the majority of preterm infants among both ART-conceived infants and all births (68% and 72%, respectively).

**TABLE 6:** Number, percentage, and proportion of infants born with use of assisted reproductive technology,* by preterm gestational age category and female patient’s reporting area of residence^†^ at time of treatment — United States and Puerto Rico, 2016

Patient’s reporting area of residence	VPTB < 32 weeks		Proportion of ART VPTB infants among all VPTB infants (%)	Early PTB <34 weeks			Late PTB 34–36 weeks			PTB <37 weeks		
	ART infants	All infants^§^		ART infants	All infants	Proportion of ART early PTB infants among all early PTB infants (%)	ART infants	All infants	Proportion of ART late PTB infants among all late PTB infants (%)	ART infants	All infants^§^	Proportion of ART PTB infants among all PTB infants (%)
	No. (%)	No. (%)		No. (%)	No. (%)		No. (%)	No. (%)		No. (%)	No. (%)	
Alabama	35 (7.9)	1,276 (2.2)	2.7	63 (14.2)	2,100 (3.6)	3	123 (27.8)	4,983 (8.4)	2.5	186 (42.0)	7,083 (12.0)	2.6
Alaska	7 (8.5)	127 (1.1)	5.5	7 (8.5)	240 (2.1)	2.9	19 (23.2)	759 (6.8)	2.5	26 (31.7)	999 (8.9)	2.6
Arizona	78 (6.9)	1,119 (1.3)	7.0	146 (13.0)	1,969 (2.3)	7.4	244 (21.7)	5,685 (6.7)	4.3	390 (34.7)	7,654 (9.1)	5.1
Arkansas	16 (7.4)	707 (1.8)	2.3	30 (13.9)	1,166 (3.0)	2.6	51 (23.6)	2,991 (7.8)	1.7	81 (37.5)	4,157 (10.9)	1.9
California	389 (4.2)	6,203 (1.3)	6.3	774 (8.4)	11,227 (2.3)	6.9	1,656 (18.0)	30,847 (6.3)	5.4	2,430 (26.4)	42,074 (8.6)	5.8
Colorado	49 (4.0)	830 (1.2)	5.9	126 (10.3)	1,612 (2.4)	7.8	282 (23.1)	4,286 (6.4)	6.6	408 (33.4)	5,898 (8.9)	6.9
Connecticut	66 (4.8)	541 (1.5)	12.2	122 (8.8)	926 (2.6)	13.2	285 (20.6)	2,449 (6.8)	11.6	407 (29.4)	3,375 (9.4)	12.1
Delaware	14 (5.1)	216 (2.0)	6.5	20 (7.4)	344 (3.1)	5.8	37 (13.6)	761 (6.9)	4.9	57 (21.0)	1,105 (10.1)	5.2
District of Columbia	— (—)^¶^	195 (2.0)	—^¶^	19 (5.5)	334 (3.4)	5.7	49 (14.1)	725 (7.4)	6.8	68 (19.6)	1,059 (10.7)	6.4
Florida	149 (4.9)	3,887 (1.7)	3.8	284 (9.4)	6,599 (2.9)	4.3	675 (22.3)	16,223 (7.2)	4.2	959 (31.6)	22,822 (10.1)	4.2
Georgia	82 (5.3)	2,659 (2.0)	3.1	132 (8.5)	4,331 (3.3)	3.0	307 (19.9)	10,246 (7.9)	3.0	439 (28.4)	14,577 (11.2)	3.0
Hawaii	42 (10.4)	296 (1.6)	14.2	66 (16.3)	489 (2.7)	13.5	85 (21.0)	1,415 (7.8)	6.0	151 (37.4)	1,904 (10.5)	7.9
Idaho	21 (7.3)	297 (1.3)	7.1	32 (11.1)	500 (2.2)	6.4	83 (28.8)	1,508 (6.7)	5.5	115 (39.9)	2,008 (8.9)	5.7
Illinois	227 (5.1)	2,765 (1.8)	8.2	429 (9.7)	4,766 (3.1)	9.0	833 (18.9)	11,186 (7.2)	7.4	1,262 (28.6)	15,952 (10.3)	7.9
Indiana	36 (4.2)	1,405 (1.7)	2.6	83 (9.8)	2,348 (2.8)	3.5	187 (22.0)	5,939 (7.1)	3.1	270 (31.7)	8,287 (10.0)	3.3
Iowa	22 (3.1)	554 (1.4)	4.0	61 (8.7)	940 (2.4)	6.5	131 (18.7)	2,712 (6.9)	4.8	192 (27.4)	3,652 (9.3)	5.3
Kansas	23 (5.3)	505 (1.3)	4.6	38 (8.7)	940 (2.5)	4.0	98 (22.5)	2,517 (6.6)	3.9	136 (31.3)	3,457 (9.1)	3.9
Kentucky	32 (6.0)	1,014 (1.8)	3.2	56 (10.6)	1,722 (3.1)	3.3	125 (23.6)	4,600 (8.3)	2.7	181 (34.2)	6,322 (11.4)	2.9
Louisiana	44 (8.3)	1,370 (2.2)	3.2	67 (12.7)	2,243 (3.6)	3.0	119 (22.5)	5,739 (9.1)	2.1	186 (35.2)	7,982 (12.6)	2.3
Maine	7 (3.6)	163 (1.3)	4.3	12 (6.3)	276 (2.2)	4.3	26 (13.5)	813 (6.4)	3.2	38 (19.8)	1,089 (8.6)	3.5
Maryland	89 (4.4)	1,346 (1.8)	6.6	156 (7.7)	2,220 (3.0)	7.0	339 (16.7)	5,188 (7.1)	6.5	496 (24.5)	7,408 (10.1)	6.7
Massachusetts	105 (3.1)	922 (1.3)	11.4	234 (7.0)	1,677 (2.4)	14.0	522 (15.6)	4,491 (6.3)	11.6	756 (22.6)	6,168 (8.6)	12.3
Michigan	105 (5.9)	1,939 (1.7)	5.4	221 (12.5)	3,290 (2.9)	6.7	452 (25.6)	8,200 (7.2)	5.5	673 (38.1)	11,490 (10.1)	5.9
Minnesota	37 (3.0)	940 (1.3)	3.9	99 (8.0)	1,592 (2.3)	6.2	304 (24.6)	4,529 (6.5)	6.7	403 (32.7)	6,121 (8.8)	6.6
Mississippi	13 (5.7)	889 (2.3)	1.5	23 (10.1)	1,527 (4.0)	1.5	49 (21.6)	3,647 (9.6)	1.3	72 (31.7)	5,174 (13.6)	1.4
Missouri	54 (5.3)	1,205 (1.6)	4.5	130 (12.8)	2,094 (2.8)	6.2	256 (25.2)	5,490 (7.3)	4.7	386 (38.1)	7,584 (10.2)	5.1
Montana	11 (7.7)	158 (1.3)	7.0	21 (14.7)	290 (2.4)	7.2	34 (23.8)	784 (6.4)	4.3	55 (38.5)	1,074 (8.7)	5.1
Nebraska	13 (4.1)	381 (1.4)	3.4	35 (11.0)	693 (2.6)	5.1	73 (23.0)	1,861 (7.0)	3.9	108 (34.0)	2,554 (9.6)	4.2
Nevada	30 (5.2)	556 (1.5)	5.4	62 (10.8)	985 (2.7)	6.3	135 (23.6)	2,773 (7.6)	4.9	197 (34.4)	3,758 (10.4)	5.2
New Hampshire	13 (4.8)	142 (1.2)	9.2	20 (7.4)	284 (2.3)	7.0	35 (13.0)	670 (5.5)	5.2	55 (20.4)	954 (7.8)	5.8
New Jersey	183 (4.6)	1,595 (1.6)	11.5	348 (8.8)	2,876 (2.8)	12.1	743 (18.7)	7,250 (7.1)	10.2	1,091 (27.5)	10,126 (9.9)	10.8
New Mexico	8 (8.2)	427 (1.7)	1.9	12 (12.4)	710 (2.9)	1.7	30 (30.9)	1,754 (7.1)	1.7	42 (43.3)	2,464 (10.0)	1.7
New York	267 (3.8)	3,388 (1.4)	7.9	490 (7.1)	5,953 (2.5)	8.2	1,291 (18.6)	15,003 (6.4)	8.6	1,781 (25.7)	20,956 (8.9)	8.5
North Carolina	89 (5.4)	2,237 (1.9)	4.0	178 (10.8)	3,835 (3.2)	4.6	331 (20.0)	8,707 (7.2)	3.8	509 (30.8)	12,542 (10.4)	4.1
North Dakota	20 (14.3)	181 (1.6)	11.0	26 (18.6)	281 (2.5)	9.3	38 (27.1)	759 (6.7)	5.0	64 (45.7)	1,040 (9.1)	6.2
Ohio	132 (6.8)	2,485 (1.8)	5.3	227 (11.6)	4,280 (3.1)	5.3	433 (22.2)	10,108 (7.3)	4.3	660 (33.8)	14,388 (10.4)	4.6
Oklahoma	23 (5.9)	796 (1.5)	2.9	42 (10.7)	1,411 (2.7)	3.0	109 (27.7)	4,186 (8.0)	2.6	151 (38.4)	5,597 (10.6)	2.7
Oregon	20 (3.1)	527 (1.2)	3.8	45 (7.0)	953 (2.1)	4.7	142 (22.1)	2,667 (5.9)	5.3	187 (29.1)	3,620 (7.9)	5.2
Pennsylvania	109 (4.6)	2,232 (1.6)	4.9	189 (8.0)	3,755 (2.7)	5.0	390 (16.6)	9,207 (6.6)	4.2	579 (24.6)	12,962 (9.3)	4.5
Puerto Rico	— (—)^¶^	587 (2.1)	—^¶^	5 (6.2)	1,140 (4.0)	0.4	23 (28.4)	2,892 (10.2)	0.8	28 (34.6)	4,032 (14.3)	0.7
Rhode Island	13 (5.7)	174 (1.6)	7.5	18 (7.9)	280 (2.6)	6.4	54 (23.7)	728 (6.7)	7.4	72 (31.6)	1,008 (9.3)	7.1
South Carolina	45 (6.2)	1,136 (2.0)	4.0	77 (10.7)	1,909 (3.3)	4.0	196 (27.1)	4,486 (7.8)	4.4	273 (37.8)	6,395 (11.2)	4.3
South Dakota	6 (4.2)	145 (1.2)	4.1	13 (9.0)	255 (2.1)	5.1	32 (22.2)	843 (6.9)	3.8	45 (31.3)	1,098 (8.9)	4.1
Tennessee	60 (8.7)	1,490 (1.8)	4.0	95 (13.7)	2,579 (3.2)	3.7	174 (25.1)	6,506 (8.1)	2.7	269 (38.8)	9,085 (11.2)	3.0
Texas	401 (6.6)	6,593 (1.7)	6.1	782 (12.9)	11,421 (2.9)	6.8	1,494 (24.6)	29,967 (7.5)	5.0	2,276 (37.5)	41,388 (10.4)	5.5
Utah	60 (5.9)	684 (1.4)	8.8	102 (10.1)	1,206 (2.4)	8.5	285 (28.1)	3,645 (7.2)	7.8	387 (38.2)	4,851 (9.6)	8.0
Vermont	6 (5.8)	74 (1.3)	8.1	14 (13.5)	134 (2.3)	10.4	18 (17.3)	323 (5.6)	5.6	32 (30.8)	457 (7.9)	7.0
Virginia	107 (5.1)	1,639 (1.6)	6.5	174 (8.3)	2,836 (2.8)	6.1	374 (17.9)	6,956 (6.8)	5.4	548 (26.3)	9,792 (9.6)	5.6
Washington	75 (5.0)	1,034 (1.1)	7.3	127 (8.5)	1,917 (2.1)	6.6	278 (18.5)	5,447 (6.0)	5.1	405 (27.0)	7,364 (8.1)	5.5
West Virginia	9 (6.9)	308 (1.6)	2.9	17 (13.1)	570 (3.0)	3.0	42 (32.3)	1,689 (8.9)	2.5	59 (45.4)	2,259 (11.8)	2.6
Wisconsin	34 (4.0)	991 (1.5)	3.4	74 (8.7)	1,757 (2.6)	4.2	184 (21.6)	4,628 (6.9)	4.0	258 (30.2)	6,385 (9.6)	4.0
Wyoming	— (—)^¶^	114 (1.5)	—^¶^	6 (8.3)	194 (2.6)	2.6	14 (23.3)	506 (6.9)	2.8	19 (31.7)	700 (9.5)	2.7
**Total**	**3,484 (5.0)**	**63,444 (1.6)**	**5.5**	**6,629 (9.5)**	**109,976 (2.8)**	**6.0**	**14,289 (20.4)**	**282,274 (7.1)**	**5.1**	**20,918 (29.9)**	**392,250 (9.9)**	**5.3**

**Abbreviations:** ART = assisted reproductive technology; PTB = preterm birth; VPTB = very preterm birth.

* ART totals include infants conceived from ART procedures performed in 2015 and born in 2016 and infants conceived from ART procedures performed in 2016 and born in 2016. Total ART births exclude births to nonresidents and include only infants with gestational age data available.

^†^ In cases of missing residency data (0.4%), the patient’s residence was assigned as the location where the ART procedure was performed.

^§^ U.S. births include births to nonresidents. **Source:** CDC Wonder [Internet]. Natality public use data 2007–2016. Atlanta, GA: US Department of Health and Human Services, CDC; 2018.

^¶^ To protect confidentiality, cells with values of 1–4 for ART infants and cells with values of 0–9 for all infants are suppressed. Also suppressed are data that can be used to derive suppressed cell values. These values are included in the totals.

The percentage of ART-conceived infants who had low birthweight was 8.7% among singletons, 54.9% among twins, and 94.9% among triplets and higher-order multiples; the corresponding percentages among all infants were 6.2% among singletons, 55.4% among twins, and 94.6% among triplets and higher-order multiples (Table 7).

**TABLE 7:** Percentage of low birthweight infants (<2,500 g) among infants born with assisted reproductive technology* and all U.S. infants, by plurality and female patient’s reporting area of residence^†^ at time of treatment — United States and Puerto Rico, 2016

Patient’s reporting area of residence	ART singleton infants (%)	All singleton infants^§^ (%)	ART twin infants^¶^ (%)	All twin infants^§^ (%)	ART triplets and higher-order infants^¶^ (%)	All triplets and higher-order infants^§^ (%)
Alabama	14.6	7.9	56.3	60.4	—**	96.7
Alaska	—^††^	4.5	62.2	42.0	—^§§^	—**
Arizona	7.8	5.6	56.3	54.5	100.0	95.9
Arkansas	4.5	7.1	64.0	57.6	—^§§^	92.6
California	8.3	5.1	52.8	53.1	96.3	96.7
Colorado	11.6	7.0	62.5	61.2	—**	95.6
Connecticut	8.4	5.5	47.6	51.6	100.0	94.1
Delaware	5.6	7.2	68.4	59.1	—^§§^	—**
District of Columbia	6.2	7.9	70.4	56.7	—**^,††^	—**
Florida	8.1	6.8	55.9	56.6	81.8	91.9
Georgia	8.6	7.7	65.0	60.8	—**	91.4
Hawaii	9.3	6.4	64.3	59.3	—**	94.1
Idaho	7.0	5.3	50.4	49.1	—**	92.9
Illinois	8.7	6.3	53.4	53.5	93.9	89.9
Indiana	9.4	6.2	48.2	55.2	—**	88.5
Iowa	6.0	4.8	44.4	54.1	—**	98.0
Kansas	9.3	5.3	48.8	52.3	—**	97.3
Kentucky	7.3	6.9	53.7	57.9	—**	98.7
Louisiana	8.8	8.3	56.0	64.5	—**	100.0
Maine	6.2	5.3	44.7	53.4	—**^,††^	—**
Maryland	9.7	6.6	54.6	55.3	—**	96.1
Massachusetts	8.3	5.5	54.6	52.5	—**	95.6
Michigan	9.0	6.4	56.8	54.5	92.3	97.1
Minnesota	7.4	4.7	50.1	50.2	—**	98.1
Mississippi	10.0	9.3	60.6	63.1	—**^,††^	97.2
Missouri	9.2	6.6	56.9	54.9	—**	98.9
Montana	7.5	6.1	65.2	55.7	—^§§^	—**
Nebraska	11.2	5.3	46.4	45.7	—^§§^	89.6
Nevada	10.2	6.6	61.2	58.5	—**^,††^	95.5
New Hampshire	5.9	4.9	53.2	49.9	—**^,††^	85.7
New Jersey	9.2	5.9	60.0	55.1	100.0	93.4
New Mexico	13.6	7.4	79.4	58.7	—**^,††^	—**
New York	8.3	5.9	52.8	54.4	94.9	96.4
North Carolina	8.9	7.2	53.9	57.1	100.0	95.9
North Dakota	12.7	4.8	60.8	52.7	—**^,††^	—**
Ohio	8.3	6.6	55.0	53.7	87.9	94.8
Oklahoma	11.8	6.1	56.7	53.4	—**	94.6
Oregon	6.9	4.7	47.6	52.4	—**	93.8
Pennsylvania	8.8	6.3	50.3	52.2	—**	92.7
Puerto Rico	11.1	8.9	71.4	64.9	—^§§^	—**
Rhode Island	10.8	5.8	46.9	55.0	—**^,††^	—**
South Carolina	9.4	7.3	50.4	58.7	88.5	94.8
South Dakota	6.8	4.9	38.7	49.7	—**	100.0
Tennessee	12.8	7.2	55.4	59.1	—**	93.4
Texas	9.5	6.5	59.4	58.3	97.9	95.5
Utah	11.9	5.0	56.9	54.9	—**	98.1
Vermont	9.7	5.3	74.2	46.5	—**^,††^	—**
Virginia	7.9	6.1	53.9	55.4	—**	96.7
Washington	8.5	4.9	53.9	50.1	100.0	93.8
West Virginia	7.6	7.8	50.0	58.1	—**^,††^	100.0
Wisconsin	6.1	5.5	46.8	52.8	—**^,††^	87.0
Wyoming	—^††^	6.7	36.8	58.8	—^§§^	—**
**Total**	**8.7**	**6.2**	**54.9**	**55.4**	**94.9**	**94.6**

**Abbreviation:** ART = assisted reproductive technology.

* ART totals include infants conceived from ART procedures performed in 2015 and born in 2016 and infants conceived from ART procedures performed in 2016 and born in 2016. Total ART births exclude births to nonresidents and only include infants with birthweight data available.

^†^ In cases of missing residency data (0.4%), the patient’s residence was assigned as the location where the ART procedure was performed.

^§^ U.S. births include births to nonresidents. **Source:** CDC Wonder [Internet]. Natality public use data 2007–2016. Atlanta, GA: US Department of Health and Human Services, CDC; 2018.

^¶^ Includes only the number of infants live born in a multiple-birth delivery. For example, if three infants were born in a live-birth delivery and one of the three infants was stillborn, the total number of live-born infants would be two. However, the two infants still would be counted as triplets.

** Estimates on the basis of N = <20 in the denominator have been suppressed because such rates are considered unstable.

^††^ To protect confidentiality, cells with values of 1–4 for ART infants and cells with values of 0–9 for all infants are suppressed. Also suppressed are data that can be used to derive suppressed cell values. These values are included in the totals.

^§§^ Estimates were not calculated because N = 0 for the denominator.

The percentage of ART-conceived infants who were born preterm was 13.7% among singletons, 64.2% among twins, and 97.0% among triplets and higher-order infants; the corresponding percentages among all infants were 7.8% among singletons, 59.9% among twins, and 97.7% among triplets and higher-order multiples (Table 8).

**TABLE 8:** Percentages of preterm (<37 weeks) infants among infants born with use of assisted reproductive technology* and all U.S. infants, by plurality and female patient’s reporting area of residence^†^ at time of treatment — United States and Puerto Rico, 2016

Patient’s reporting area of residence	ART singleton infants (%)	All singleton infants^§^ (%)	ART twin infants^¶^ (%)	All twin infants^§^ (%)	ART triplets and higher-order infants^¶^ (%)	All triplets and higher-order infants^§^ (%)
Alabama	19.1	9.4	70.9	64.5	—**	100.0
Alaska	11.1	7.2	56.8	50.3	—^§§^	—**
Arizona	13.5	7.2	65.2	58.0	100.0	100.0
Arkansas	11.3	8.9	79.5	66.5	—^§§^	100.0
California	12.7	6.8	59.9	55.8	100.0	99.5
Colorado	15.2	7.0	71.0	58.1	—**	95.6
Connecticut	12.6	7.0	58.4	53.5	100.0	100.0
Delaware	10.7	8.2	84.2	61.2	—^§§^	—**
District of Columbia	10.6	8.6	65.4	54.9	—**^,††^	—**
Florida	13.9	8.2	64.1	59.7	100.0	95.9
Georgia	14.0	9.0	68.0	63.4	—**	98.0
Hawaii	17.9	8.2	63.9	65.9	—**	97.1
Idaho	15.2	7.0	74.8	57.9	—**	89.3
Illinois	13.5	8.0	60.1	60.2	93.9	97.6
Indiana	15.1	7.8	58.0	60.8	—**	94.7
Iowa	13.5	7.1	59.4	63.0	—**	98.0
Kansas	16.4	7.2	64.0	60.0	—**	100.0
Kentucky	15.0	8.9	67.9	66.5	—**	100.0
Louisiana	15.9	10.1	69.2	70.1	—**	100.0
Maine	8.8	6.8	52.4	55.5	—**^,††^	—**
Maryland	13.7	7.9	63.3	61.6	—**	100.0
Massachusetts	12.2	6.5	61.8	56.7	—**	96.7
Michigan	13.4	7.8	65.9	60.3	100.0	97.8
Minnesota	11.9	6.7	66.8	58.5	—**	100.0
Mississippi	14.0	11.3	70.1	70.3	—**^,††^	91.7
Missouri	15.3	7.9	73.7	61.0	—**	98.9
Montana	11.4	7.0	71.9	56.5	—^§§^	—**
Nebraska	17.5	7.6	64.3	55.3	—^§§^	89.6
Nevada	14.3	8.3	75.1	63.8	—**^,††^	100.0
New Hampshire	9.8	6.1	51.6	54.4	—**^,††^	95.2
New Jersey	13.9	7.5	65.4	59.5	100.0	100.0
New Mexico	19.0	8.3	77.8	63.9	—**^,††^	—**
New York	12.5	6.8	57.9	55.2	82.5	97.3
North Carolina	13.5	8.2	60.0	59.8	100.0	97.6
North Dakota	17.5	6.9	67.6	66.5	—**^,††^	—**
Ohio	12.4	8.2	63.8	59.3	100.0	100.0
Oklahoma	16.6	8.6	67.1	62.8	—**	97.3
Oregon	10.7	6.1	56.7	54.4	—**	100.0
Pennsylvania	12.6	7.3	57.7	56.9	—**	99.1
Puerto Rico	10.9	12.9	65.7	66.0	—^§§^	—**
Rhode Island	14.5	6.9	69.7	61.6	—**^,††^	—**
South Carolina	14.3	8.8	66.2	62.8	100.0	96.1
South Dakota	9.7	6.9	43.3	53.8	—**	100.0
Tennessee	19.8	9.0	73.6	65.2	—**	100.0
Texas	17.0	8.3	74.2	63.0	94.1	96.5
Utah	18.1	7.1	67.1	64.3	—**	100.0
Vermont	12.7	6.5	66.7	44.3	—**^,††^	—**
Virginia	13.0	7.4	61.2	59.7	—**	100.0
Washington	11.8	6.4	67.7	56.8	100.0	98.4
West Virginia	27.2	9.9	73.9	62.1	—**^,††^	100.0
Wisconsin	12.5	7.5	60.3	60.9	—**^,††^	96.3
Wyoming	17.1	7.5	63.2	63.8	—^§§^	—**
**Total**	**13.7**	**7.8**	**64.2**	**59.9**	**97.0**	**97.7**

**Abbreviation:** ART = assisted reproductive technology.

* ART totals include infants conceived from ART procedures performed in 2015 and born in 2016 and infants conceived from ART procedures performed in 2016 and born in 2016. Total ART births exclude births to nonresidents and includes only infants with gestational age data available.

^†^ In cases of missing residency data (0.4%), the patient’s residence was assigned as the location where the ART procedure was performed.

^§^ U.S. births include births to nonresidents. **Source:** Martin JA, Hamilton BE, Osterman MJ, Driscoll AK, Mathews TJ. Births: final data for 2016. Natl Vital Stat Rep 2018;67:1–55.

^¶^ Includes only the number of infants live born in a multiple-birth delivery. For example, if three infants were born in a live-birth delivery and one of the three infants was stillborn, the total number of live-born infants would be two. However, the two infants still would be counted as triplets.

** Estimates on the basis of N = <20 in the denominator have been suppressed because such rates are considered unstable.

^††^ To protect confidentiality, cells with values of 1–4 for ART infants and cells with values of 0–9 for all infants are suppressed. Also suppressed are data that can be used to derive suppressed cell values. These values are included in the totals.

^§§^ Estimates were not calculated because N = 0 for the denominator.

The percentage of ART-conceived SGA singletons was 8.7% for infants born preterm (<37 weeks), 8.0% for full term infants (37–41 weeks), and 8.1% overall (22–44 weeks); the corresponding percentages among all singleton infants were 9.3%, 10.5%, and 9.9%. Nationally, ART-conceived SGA singleton infants contributed to 1.0% of all SGA singleton infants (Table 9).

**TABLE 9:** Percentages and proportions of small for gestational age singleton infants born with use of assisted reproductive technology,* by gestational age and female patient’s reporting area of residence^†^ at time of treatment — United States and Puerto Rico, 2016

Patient’s reporting area of residence	ART singleton infants			All singleton infants^§^			Proportion of ART SGA singleton infants among all SGA singleton infants (%)
	PTB <37 weeks (% SGA)	FT 37–41 weeks (% SGA)	All 22–44 weeks (% SGA)	PTB <37 weeks (% SGA)	FT 37–41 weeks (% SGA)	All 22–44 weeks (% SGA)	
Alabama	—^¶^	9.8	9.5	9.1	12.8	11.7	0.4
Alaska	0.0	13.2	11.9	7.7	6.4	6.1	0.8
Arizona	7.7	10.0	9.9	8.3	9.8	9.1	0.9
Arkansas	—^¶,^**	—^¶^	4.5	8.3	11.8	10.7	0.2
California	6.7	9.1	8.9	10.2	9.4	9.2	1.3
Colorado	10.8	10.4	10.4	11.3	13.7	12.9	1.0
Connecticut	4.5	8.9	8.4	10.0	9.5	9.1	2.4
Delaware	—^¶^	4.3	4.3	9.2	10.6	9.9	1.0
District of Columbia	—^¶^	6.6	6.6	9.9	13.4	12.2	1.7
Florida	8.5	7.9	7.9	8.9	11.5	10.6	0.7
Georgia	10.3	5.6	6.2	9.7	13.0	11.9	0.5
Hawaii	—^¶^	10.1	10.2	7.9	12.3	11.1	1.2
Idaho	0.0	9.7	8.2	8.4	9.3	8.7	0.7
Illinois	11.7	8.6	9.0	9.4	10.1	9.5	1.9
Indiana	13.6	8.5	9.4	10.1	10.2	9.8	0.6
Iowa	—^¶^	6.6	6.3	8.0	7.7	7.3	1.1
Kansas	14.6	6.5	7.8	8.4	8.0	7.9	0.8
Kentucky	—^¶^	8.0	7.1	8.5	10.5	9.7	0.5
Louisiana	—^¶^	6.7	6.5	8.6	12.4	11.3	0.3
Maine	—^¶,^**	4.7	4.9	7.6	8.8	8.2	0.7
Maryland	12.5	6.3	7.1	9.8	10.4	9.9	1.6
Massachusetts	8.4	8.7	8.7	11.0	9.6	9.2	3.6
Michigan	9.6	8.3	8.5	9.6	10.7	10.0	0.7
Minnesota	6.5	7.4	7.3	7.6	8.2	7.6	1.1
Mississippi	—^¶^	3.8	6.4	9.6	14.3	12.9	0.2
Missouri	11.2	8.1	8.7	9.0	9.9	9.4	0.8
Montana	0.0	—^¶^	—^¶^	7.7	10.5	9.7	—^¶^
Nebraska	—^¶^	4.8	5.4	8.2	8.1	7.7	0.6
Nevada	11.1	8.2	8.5	8.8	12.2	11.1	0.8
New Hampshire	—^¶,^**	7.7	8.4	9.8	9.1	8.6	1.7
New Jersey	7.5	8.8	8.7	9.5	10.5	9.9	2.6
New Mexico	0.0	—^¶^	—^¶^	10.7	13.3	12.3	—^¶^
New York	9.5	8.3	8.4	10.0	11.2	10.5	1.7
North Carolina	7.2	8.5	8.3	9.4	11.6	10.6	0.7
North Dakota	0.0	—^¶^	—^¶^	8.2	7.4	7.1	—^¶^
Ohio	7.7	8.0	8.0	9.2	10.4	9.8	0.7
Oklahoma	—^¶^	8.5	8.8	6.7	9.5	8.7	0.5
Oregon	12.2	5.2	5.9	8.4	8.4	7.9	0.7
Pennsylvania	8.2	9.1	9.0	10.3	10.6	10.1	1.1
Puerto Rico	0.0	20.0	17.8	9.2	15.6	14.2	0.2
Rhode Island	—^¶^	14.2	13.5	8.6	10.4	9.7	2.1
South Carolina	—^¶^	7.5	7.2	9.5	11.2	10.4	0.5
South Dakota	0.0	7.7	6.9	6.4	7.8	7.3	0.6
Tennessee	9.0	5.6	6.3	10.2	10.9	10.5	0.3
Texas	7.2	6.5	6.6	8.4	10.8	10.2	0.7
Utah	15.0	7.4	8.9	8.0	9.0	8.5	1.3
Vermont	—^¶,^**	10.2	11.4	8.1	9.3	8.6	1.7
Virginia	10.4	7.0	7.4	10.0	10.5	9.9	1.2
Washington	11.6	7.5	7.9	8.5	8.7	8.1	1.2
West Virginia	0.0	12.3	10.1	9.3	11.3	10.5	0.4
Wisconsin	11.5	3.2	4.2	9.4	8.6	8.2	0.4
Wyoming	0.0	21.2	17.5	10.1	12.7	11.9	0.8
**Total**	**8.7**	**8.0**	**8.1**	**9.3**	**10.5**	**9.9**	**1.0**

**Abbreviations:** ART = assisted reproductive technology; FT = full term; PTB = preterm birth; SGA = small for gestational age.

* ART totals include infants conceived from ART procedures performed in 2015 and born in 2016 and infants conceived from ART procedures performed in 2016 and born in 2016. Total ART births exclude births to nonresidents and includes only infants with gestational age data available.

^†^ In cases of missing residency data (0.4 %), the patient's residence was assigned as the location in which the ART procedure was performed.

^§^ U.S. births include births to nonresidents. **Source:** Martin JA, Hamilton BE, Osterman MJ, Driscoll AK, Mathews TJ. Births: final data for 2016. Natl Vital Stat Rep 2018;67:1–55.

^¶^ To protect confidentiality, cells with values of 1–4 for ART infants and cells with values of 0–9 for all infants are suppressed. Also suppressed are data that can be used to derive suppressed cell values. These values are included in the totals.

** Estimates on the basis of N = <20 in the denominator have been suppressed because such rates are considered unstable.

## Discussion

### Overview

The use of ART has increased substantially in the United States since the beginning of ART surveillance. In 1996 (the first full year for which ART data were reported to CDC), 20,597 infants were born from 64,036 ART procedures performed by 302 reporting clinics (*28*). Since then, the number of procedures reported to CDC and the number of infants born from ART procedures have more than tripled.

Multiple changes can be observed in ART use and outcomes by comparing reporting years 2015 (*29*) and 2016. The rate of ART use, as measured by procedures performed per 1 million women of reproductive age, increased from 2,832 to 3,075. Among women aged <35 years, the average number of embryos transferred decreased from 1.6 to 1.5, and the national eSET rate increased from 34.7% to 42.7%. Overall, the percentage of twins among ART-conceived infants decreased from 33.9% to 30.4%, and the percentage of triplets and higher-order infants decreased from 1.4% to 1.1%. The proportion of low birthweight among ART-conceived infants decreased from 25.5% to 23.6%, and preterm birth rates decreased from 31.2% to 29.9%. The contribution of ART-conceived twins to all twins born in the United States decreased from 16.8% to 16.2%. The contribution of ART-conceived infants to all triplets and higher-order infants decreased from 22.2% to 19.4%.

The contribution of ART to rates of multiple births and poor birth outcomes (low birthweight, preterm birth, and SGA) remains substantial. In 2016, the multiple birth rate was approximately nine times higher among ART-conceived infants compared with all infants (31.5% versus 3.4%), and although infants conceived with ART accounted for approximately 1.8% of total births in the United States, the proportion of multiple-birth infants attributable to ART was 16.4%.

ART-conceived twins accounted for approximately 96.5% (21,455 of 22,233) of all ART-conceived infants born in multiple-birth deliveries. Although declining, on average, 1.5 embryos were transferred among women aged <35 years, even though single-embryo transfers have been associated with better perinatal outcomes among the majority of women in this age group (*30*,*31*). The percentage of infants with low birthweight and born preterm was substantially higher among ART-conceived infants (23.6% and 29.9%, respectively) than among all infants (8.2% and 9.9%, respectively). Similar to births among the general population, ART-conceived twins and triplets and higher-order infants were more likely than singletons to be born preterm (4.7 times and 7.0 times, respectively) and with low birthweight (6.3 times and 11.0 times respectively). Nationally, the rate of preterm birth among ART singletons was approximately 1.7 times the preterm birth rate among all infants. Because ART infants are more likely to be multiples than infants among the general population, their contribution to adverse effects such as preterm birth and low birthweight continue to be noteworthy.

Comparable data on ART use and embryo transfer practices from 39 European countries (14 countries reported data from all clinics, whereas the remaining countries only reported partial data from some clinics) indicate that in 2014, ART use as defined by the number of procedures performed per 1 million women of reproductive age was 7,623; this was approximately three times higher than the rate of 2,647 in the United States in 2014 (*32*,*33*). Percentages of single-embryo transfers, which approximate eSET rates, varied across Europe, and certain countries reported single-embryo transfer rates >50% (*32*). The ART twin rate was lowest in Sweden (4.2%), which had the highest proportion of single-embryo transfers (79.9%) (*32*). Overall, approximately 82.5% of all IVF deliveries were singleton deliveries in these countries compared with 77.7% in the United States (*32*,*33*). In multiple countries in Europe, >5% of all infants born in 2014 were conceived using ART compared with 1.6% in the United States (*32*,*33*).

### Variations in ART Use by Reporting Area

ART use (as measured by the number of ART procedures performed per 1 million women of reproductive age) varied across reporting areas. Residents of 14 reporting areas (Connecticut, Delaware, Hawaii, Illinois, Maryland, Massachusetts, New Hampshire, New Jersey, New York, Pennsylvania, Rhode Island, Utah, Virginia, and the District of Columbia) had higher rates of ART use than the national rate. Although the measure of ART use is based on a denominator of women aged 15–44 years, and certain women who used ART were aged >44 years, the measure overestimates actual ART use among women aged 15–44 years but is still a useful indicator of ART use in each reporting area. Residents of California, Illinois, Massachusetts, New Jersey, New York, and Texas accounted for 46.5% of all infants conceived with ART. The large number of ART procedures performed in these six states is a result of the size of the general population (e.g., California and Texas) and high rates of ART use per capita (e.g., Massachusetts, Illinois, New Jersey, and New York).

The contribution of ART to all infants born varied substantially, even among states where a high number of ART procedures were performed. State-level differences might be explained in part by variations in health insurance coverage. Sixteen states (Arkansas, California, Connecticut, Delaware, Hawaii, Illinois, Louisiana, Maryland, Massachusetts, Montana, New Jersey, New York, Ohio, Rhode Island, Texas, and West Virginia) have passed legislation mandating that private insurers provide coverage for some fertility treatments, although not all mandates require coverage for ART. Nine states (Arkansas, Connecticut, Delaware, Hawaii, Illinois, Maryland, Massachusetts, New Jersey, and Rhode Island) have insurance mandates that cover at least one ART cycle. Seven states (California, Louisiana, Montana, New York, Ohio, Texas, and West Virginia) have insurance mandates that exclude IVF coverage. Information is available at https://resolve.org/what-are-my-options/insurance-coverage. Mandates from four of these states (Illinois, Massachusetts, New Jersey, and Rhode Island) include comprehensive coverage for at least four oocyte retrievals. Three of the four states with comprehensive mandates (Illinois, Massachusetts, and New Jersey) had rates of ART use that were at least 50% higher than the national rate. Insurance mandates for infertility treatments have been associated with greater use of ART (*34*–*36*). Other possible contributors to differences in ART use across states might include other factors affecting access to fertility clinic services and factors affecting reproductive choices.

### Elective Single-Embryo Transfer Rates

Recommendations issued by the American Society of Reproductive Medicine (ASRM) and SART on the number of embryos to transfer have been revised multiple times to reduce the likelihood of higher-order multiple deliveries (*37*–*41*). Guidance issued by ASRM and SART in 2017 focused on promoting single-embryo transfer and decreasing all multiple pregnancies, including twin gestations. Recommendations for single-embryo transfer were expanded to patients of any age transferring a euploid (i.e., chromosomally normal) embryo, selected with the assistance of preimplantation genetic screening, and for patients aged <38 years with any one of these criteria: 1) availability of quality embryos for cryopreservation, 2) history of live birth after an IVF procedure, 3) availability of vitrified blastocyst stage embryos, or 4) undergoing first frozen embryo transfer (*42*). Results of an analysis of ART cycles conducted in 2015 suggested that approximately half of ART-related multiple births resulted from the transfer of two fresh embryos among women aged <35 years and patients who received donor oocytes; therefore, reducing the number of embryos transferred from two to one among patients who have a good chance of pregnancy and live birth with single-embryo transfers will lower rates of ART-conceived twins (*43*,*44*).

Among women aged <35 years, the percentage of eSET procedures varied across reporting areas (range: 8.3%–83.9%), and remained less than the national eSET rate of 42.7% in 32 of the 52 reporting areas. Similarly, eSET rates were lower than the national eSET rate in 39 of the 52 reporting areas among women aged 35–37 years. Nonetheless, from 2009 to 2016, the national rate of eSET increased nearly six times (from 7.4% to 42.7%) among women aged <35 years (*45*). An increase was observed annually among all age groups since 2009 (when eSET rates were first reported nationally and by state), but was notable among women aged <35 years, especially between 2013 and 2014, when an historically large annual increase (33.0%) in the national eSET rate was observed (*33*). The large increase in the use of eSET at U.S. clinics during 2013–2014 might have been in response to increasing calls for promoting single-embryo transfers among younger patients with favorable prognoses by researchers worldwide as well as by the ASRM and SART Practice Committees (*31*,*41*). However, the national eSET rate in the United States is still lower than in countries that impose restrictions on the number of embryos transferred and provide public funding for ART services (ranging from two to six publicly funded cycles in certain countries) (*46*).

Certain factors might influence eSET rates, such as the patient’s age, types of infertility diagnosis, and treatment costs (*34*). Out-of-pocket costs per IVF attempt are estimated to be between $10,000 and $15,000 (*35*). In the United States, even where state-mandated, coverage for infertility treatment can vary in scope, with ART services often excluded or restricted to certain age groups or diagnoses (*34*,*36*). Furthermore, insurance mandates for infertility do not apply to employers that self-insure. In three of the four states with mandatory comprehensive insurance coverage for ART, the eSET rate among women aged <35 years was higher than the national eSET rate of 42.7% (73.4% in Massachusetts, 50% in New Jersey, and 60% in Rhode Island). The fourth state, Illinois, had an eSET rate of 39.1%. Insurance coverage for infertility treatment and enhanced coverage for ART might increase the use of eSET because patients might be more willing to transfer fewer embryos when the financial burden of treatment is diminished (*35*,*47*,*48*). Expanded insurance coverage for ART services might help support greater use of eSET and might improve adherence to professional guidance on embryo transfer practices (*43*,*44*,*48*,*49*).

### ART Multiple-Birth Infants

Singleton live-birth deliveries have lower risks than multiple-birth deliveries for adverse birth outcomes, such as prematurity, low birthweight, developmental disability, and death (*50*–*52*). To optimize healthy birth outcomes, the transfer of fewer embryos should be encouraged where appropriate, taking into consideration the patient’s age and prognosis (*30*,*42*). The percentage of multiple births among ART-conceived infants in the United States decreased from 53.1% in 2000 (when national multiple birth rates were first reported) to 31.5% in 2016 (*53*). A substantial decrease was noted for both the percentage of ART-conceived triplets and higher-order infants (from 8.9% in 2000 to 1.1% in 2016) and the percentage of ART-conceived twins (from 44.2% in 2000 to 30.4% in 2016). Certain states with the highest eSET rates (i.e., Delaware, the District of Columbia, and Massachusetts) also had the lowest rates of ART-conceived infants born in multiple births.

Until 2013, the slow decrease in twin-infant birth rates among women who undergo ART procedures was largely attributable to small but gradual increases in eSET rates (*43*,*44*). Since 2013, increases in eSET rates among women have been substantial (approximately 100% increase in women aged <35 years and 65% in women aged 35–37 years). These increases have been reflected in the decrease in twin rates (approximately 23%) and preterm birth rates (approximately 11%). In 2016, ART-conceived twins still accounted for 30% of all ART-conceived infants. On average, more than one embryo was transferred among patients aged <35 years, and almost two embryos were transferred among women aged 35–37 years who also might be good candidates for single-embryo transfers.

Transferring two embryos is associated with a slight increase in overall birth rate but a greater increase in the twin birth rate compared with transferring a single embryo (*30*,*54*). However, transferring two embryos sequentially (single-embryo transfer over two sequential procedures) has similar cumulative live-birth rates and lower twin delivery rates than transferring two embryos in a single procedure and might be a cost-effective approach, where estimated costs include ART treatment and pregnancy- and infant-associated medical costs (*55*–*57*). Evidence from other countries suggests that insurance coverage for ART combined with restrictions on the number of embryos transferred per cycle can encourage eSET procedures and reduce multiple births (*46*).

High rates of ART-conceived twins might also be partially explained by the desire for more than one child among couples experiencing infertility and their perception that the benefits of a multiple-gestation pregnancy (compared with no pregnancy) outweigh the risks (*58*–*60*). Therefore, understanding the perspective of couples undergoing infertility treatments regarding multiple-gestation pregnancies and multiple births is important. The use and acceptance of eSET among younger patients with favorable prognoses might be improved through patient education (*61*,*62*). Patient education focusing on maternal and perinatal morbidity and mortality, and the economic costs of twin gestations, has been effective in reducing the preference for twins among patients (*61*–*64*).

### ART Low Birthweight Infants, Preterm Births, and Small for Gestational Age Infants

In the United States, although rates of ART-conceived preterm and low birthweight infants have been declining steadily, the percentage of infants born with low birthweight and preterm was higher among ART-conceived infants than among all infants (*29*,*33*). Further, preterm and low birthweight rates also varied substantially across states among ART-conceived infants. For example, the percentage of ART-conceived infants born in gestational weeks 34–36 varied by state from 13.0% to 32.3%. Less variation by state was observed among all infants in the same gestational week (34–36 weeks) category (range: 5.5%–10.2%).

Fertility treatments, both ART and non-ART, contribute substantially to preterm births (*51*,*65*). Preterm births are a leading cause of infant mortality and morbidity, and preterm infants are at increased risk for death and have more health and developmental problems than full-term infants (*51*,*65*–*68*). The health risks associated with preterm birth have contributed to increased health care costs. In 2005, the societal economic cost associated with all preterm births in the United States was estimated at $26 billion annually ($51,600 per infant born preterm) (*51*). In 2012, the societal economic cost associated with ART-conceived preterm infants in the United States was estimated at approximately $1.3 billion (*69*).

Furthermore, the economic costs of multiple births underscore the importance of efforts to reduce ART-related multiple births, which in turn would reduce preterm-birth infants. In 2013, the mean health care costs to patients and insurers were estimated at $26,922 for ART-conceived singleton deliveries, $115,238 for ART-conceived twin deliveries, and $434,668 for ART-conceived triplets and higher-order infants (*70*).

In addition to the known risks for multiple births associated with ART, even singleton infants conceived with ART procedures might be at increased risk for low birthweight and preterm delivery compared with infants born among the general population. Nationally, the percentages of infants who were low birthweight (8.7%) and preterm (13.7%) were higher among ART singletons compared with all singletons (6.2% and 7.8%, respectively); however, among singletons, SGA rates were lower among ART-conceived infants who were born preterm and full term as well as among all gestational age categories (22–44 weeks) compared with all infants. This finding might be partially explained by the link between socioeconomic status and birth outcomes because ART patients tend to be more affluent and might be indicative of better health care and pregnancy monitoring for ART patients (*71*).

A previous study similarly found that the risk for SGA infants was lower among ART-conceived singletons from cryopreserved embryo transfers compared with singleton infants who were not conceived using ART (*71*). However, the same results were not seen among ART singletons conceived from fresh embryo transfers. An older study also found that ART-conceived infants, including singletons, were at higher risk for SGA than those who were conceived without use of any fertility treatment (*72*). Another study found that SGA risks among singletons varied between ART births using donor oocytes compared with those using autologous oocytes (*73*). Although low birthweight is a risk factor for adverse effects among newborns and is usually associated with preterm births, SGA might be a better indicator of these risk factors among newborns than low birthweight because it accounts for gestational age. More research is needed to better understand the risk for SGA among ART-conceived infants as well as the extent to which SGA can vary by type of ART cycle performed.

ART only partially explains the overall prevalence of these adverse outcomes in the United States. Other factors influencing multiple births include advanced maternal age at conception and the use of non-ART fertility treatments (*51*,*65*,*74*,*75*). During 1980–2009, older age of women giving birth accounted for a substantial increase in twins, thought to be attributed to the increased likelihood of an embryo splitting as a woman ages (*74*). The risk for multiple gestations associated with non-ART fertility treatments (i.e., controlled ovarian stimulation and ovulation induction coupled with timed intercourse or intrauterine insemination) is less well documented than that associated with ART procedures because clinics are only required to report data on ART use. However, research suggests that non-ART fertility treatments might contribute to a larger percentage of multiple births than ART fertility treatments. In 2015, approximately 17% of multiple-birth infants in the United States were attributable to IVF fertility treatments, whereas 29% were attributable to non-IVF fertility treatments (*76*).

Further efforts are needed to monitor the use of non-ART fertility treatments and their role in multiple-birth deliveries, particularly because the ability to control the occurrence of a multiple birth is more challenging when using non-ART fertility treatments (*51*,*65*). Multiple gestations resulting from non-ART fertility treatments also contribute to preterm births and low birthweight (*51*,*65*). Additional research is needed to identify the causes and consequences of preterm births that occur specifically as a result of infertility treatments and to support further guidance to reduce the number of multiple gestations (*51*,*65*). CDC is monitoring the prevalence of ART and non-ART fertility treatment use and resultant outcomes among women who had live births in certain states participating in the Pregnancy Risk Assessment Monitoring System (*77*–*79*).

## Limitations

The findings in this report are subject to at least seven limitations. First, ART surveillance data were reported for each ART procedure performed rather than for each patient who used ART. As a result, because patients can achieve a successful pregnancy after undergoing multiple procedures, the procedure-specific success rates reported here might underestimate the actual per-patient success rates. Second, preterm birth, low birthweight, and SGA infants could be associated with factors contributing to underlying infertility or other maternal or parental factors and not necessarily to ART procedures. Third, approximately 8% of fertility clinics that performed ART in 2016 did not report their data to CDC, and these clinics might have had results differing from reporting clinics. Fourth, the measure of ART use is based on a denominator of women aged 15–44 years but a numerator of all procedures performed among women using ART, and therefore might be higher than the actual ART use rate among women in the United States who used ART to conceive. Fifth, in 2014 the methods for estimating gestational age for women who did not undergo ART procedures changed from last normal menstrual period (LMP) measures to obstetric estimate of gestation at delivery (OE)–based measures. The OE-based preterm birth rates are lower than those estimated with LMP, and therefore comparisons with previous years should be made with caution. Sixth, comparisons between ART births and U.S. births should be made with caution because ART births exclude births to non-U.S. residents that are included in U.S. births. Finally, the number of ART procedures reported to CDC in 2016 included procedures with frozen eggs that were thawed with the intent to transfer, and therefore comparisons with previous years in which procedures using thawed eggs were excluded from analyses should be made with caution.

## Conclusion

Since 1995, the number of ART procedures performed in the United States and the number of infants born as a result of these procedures have more than tripled. With this increasing use, ART-conceived infants represented approximately 2% of infants born in the United States in 2016 and contributed to the prevalence of low birthweight and preterm deliveries. Furthermore, among ART-conceived infants, although the percentage of triplets or higher-order infants has decreased since 2000, the percentage of twins has remained at more than 30% despite declining trends. In 2016, approximately one third of ART-conceived infants were multiple-birth infants. Because of higher rates of preterm birth and low birthweight among multiple-birth infants, the impact of ART on poor birth outcomes remains substantial. This report provides information that allows state health departments to monitor the extent of ART-related adverse perinatal outcomes among singletons, twins, and triplets and higher-order infants in their reporting areas and take action to initiate programs and policies to reduce the adverse effects of ART multiple births.

Comprehensive insurance coverage of ART can help increase access to fertility treatments. More research is needed to ascertain the influence of state health insurance mandates on ART use, embryo transfer practices, infant outcomes, and economic and out-of-pocket patient costs of multiple births (*31*,*43*,*44*,*80*).

Addressing the risk for multiple-birth deliveries also requires understanding the perspectives of couples undergoing infertility treatments who might view a multiple birth, especially twins, as an acceptable or desired outcome or who might lack awareness of the increased risks associated with multiple births to mothers and infants. Although the majority of clinicians acknowledge that the birth of a healthy singleton is the best outcome of ART, they might be sensitive to patient perspectives and experiences with infertility (*37*,*80*). Clinicians need to be aware of ongoing efforts and new guidance published in 2017 (*42*) to limit the number of embryos transferred and reduce the rate of multiple births, particularly twins. The wider implementation of eSET, when clinically appropriate, should be encouraged as a mechanism of promoting singleton infant births among ART pregnancies (*30*,*42*,*44*).

In 2014, CDC outlined a public health strategy for the detection, prevention, and management of infertility, including improving ART practice and outcomes, through coordinated efforts of government and nongovernment organizations (*81*). Of public health importance is the role that infertility treatment might have on adverse birth outcomes, primarily because of higher rates of multiple births.

As of January 2016, all states had adopted the 2003 revision of the birth certificate that includes information on whether the pregnancy resulted from the use of infertility treatment; 47 states and the District of Columbia differentiate between the use of ART and non-ART treatments. CDC also has been working to improve state-based surveillance for ART, infertility, and other birth-related matters by linking data from NASS to data collected by states (i.e., birth certificate, infant death, hospital discharge, and birth defect registry information). This initiative, the States Monitoring Assisted Reproductive Technology (SMART) Collaborative (https://www.cdc.gov/art/smart/index.html), has been determined to be feasible and useful for monitoring long-term outcomes of ART in selected states (*79*,*82*). CDC will continue to provide updates of ART use in the United States as data become available. A state-specific website that presents key ART statistics can be found at https://www.cdc.gov/art/state-specific-surveillance/index.html.
